# Timing of implicit processes in aphasia: An event-related potential investigation of masked priming effects

**DOI:** 10.1016/j.clinph.2025.2110972

**Published:** 2025-08-12

**Authors:** Ashlie H. Pankonin, JoAnn P. Silkes

**Affiliations:** a SDSU-UCSD Joint Doctoral Program in Language and Communicative Disorders, School of Speech, Language, and Hearing Sciences, San Diego State University, Department of Cognitive Science, University of California, San Diego, 5500 Campanile Rd., SLHS-1518, San Diego, CA, USA 92182-1518; b Department of Speech and Hearing Sciences, University of Washington, 1417 NE 42nd Street, Seattle, WA, USA 98105-6246; c School of Speech, Language, and Hearing Sciences, San Diego State University, 5500 Campanile Rd., SLHS-1518, San Diego, CA, USA 92182-1518

**Keywords:** Aphasia, Anomia, ERPs, Priming, Masked Priming, Automatic spreading activation

## Abstract

**Objectives:**

The mechanisms responsible for anomia in aphasia are not well understood. This study explores the idea that anomia could be due to changes in the timing of automatic spreading activation in the lexical system.

**Methods:**

Fourteen people with aphasia (PWA) and 13 control adults completed a masked priming event-related potential (ERP) task. Target words were preceded by masked, visible, or no primes and were either immediate or delayed repetitions of those primes. Prime-target intervals were varied. Analyses primarily focused on N400 effects.

**Results:**

PWA showed N400 priming effects for both immediate and delayed repetitions of masked primes, but control adults only showed priming effects for immediate repetitions of masked primes. Additionally, PWA’s priming effects were equal for delayed repetitions of masked and visible primes while the control adults showed greater priming effects for delayed repetitions of visible than masked primes.

**Conclusions:**

Findings suggest PWA experience prolonged lexical activation and might be less able to integrate implicit and explicit information than control adults.

**Significance:**

Improved understanding of the underlying mechanisms of language impairment in aphasia will support development of more efficient, effective aphasia treatment methods.

## 1. Introduction

Aphasia is an acquired language disorder, generally resulting from a stroke in the left hemisphere of the brain. People with aphasia (PWA) have problems speaking, listening, reading, and writing. The severity and balance of impairment across modalities differs between people, but the one ubiquitous symptom of aphasia is *anomia*, or difficulty retrieving words (Benson, 1988; Goodglass & Wingfield, 1997; Laine & Martin, 2013; Raymer, 2018). As a result, aphasia treatment often focuses on improving word retrieval (Nickels, 2002; Raymer & Roitsch, 2023). To date, however, there is still no clear understanding of the mechanism(s) responsible for this particular impairment, which means treatment methods cannot be maximally effective. The study described here is designed to improve our understanding of the mechanisms behind word retrieval problems in aphasia using event-related potentials (ERPs), with the ultimate goal of informing improvement of treatment methods.

### 1.1. Language processing in aphasia

Word retrieval problems in aphasia are generally understood to reflect impaired access to, rather than a loss of, linguistic information. This view is supported by evidence that aphasia can be transient (e.g., Chu et al., 2014; Kimura et al., 2003; Riecker et al., 2004; Wilson et al., 2015) and that language performance can be highly variable within an individual (e.g., Cameron et al., 2010; Creet et al., 2019; Duncan et al., 2016; Galletta & Goral, 2018; Tseng et al., 1993). One proposed mechanism for this impaired access is an alteration in the timing of *automatic spreading activation* within the language network (Dell et al., 1997; Ferrill et al., 2012; Prather, 1994; Prather et al., 1997; Schwartz et al., 1994; Silkes et al., 2020; Silkes & Rogers, 2012).

In network models of language processing, such as the Interactive Activation (e.g., Dell, 1986; Dell & O’Seaghdha, 1992) and Parallel Distributed Processing (e.g., Nadeau, 2001) models, automatic spreading activation is responsible for the implicit activation and integration of semantic, lexical, phonological, and orthographic representations. In a typical system, the strength of connections between representations varies, and the rapid spread and decay of activation enable language processing, including word retrieval, to proceed without interference.

**Fig. 1.**
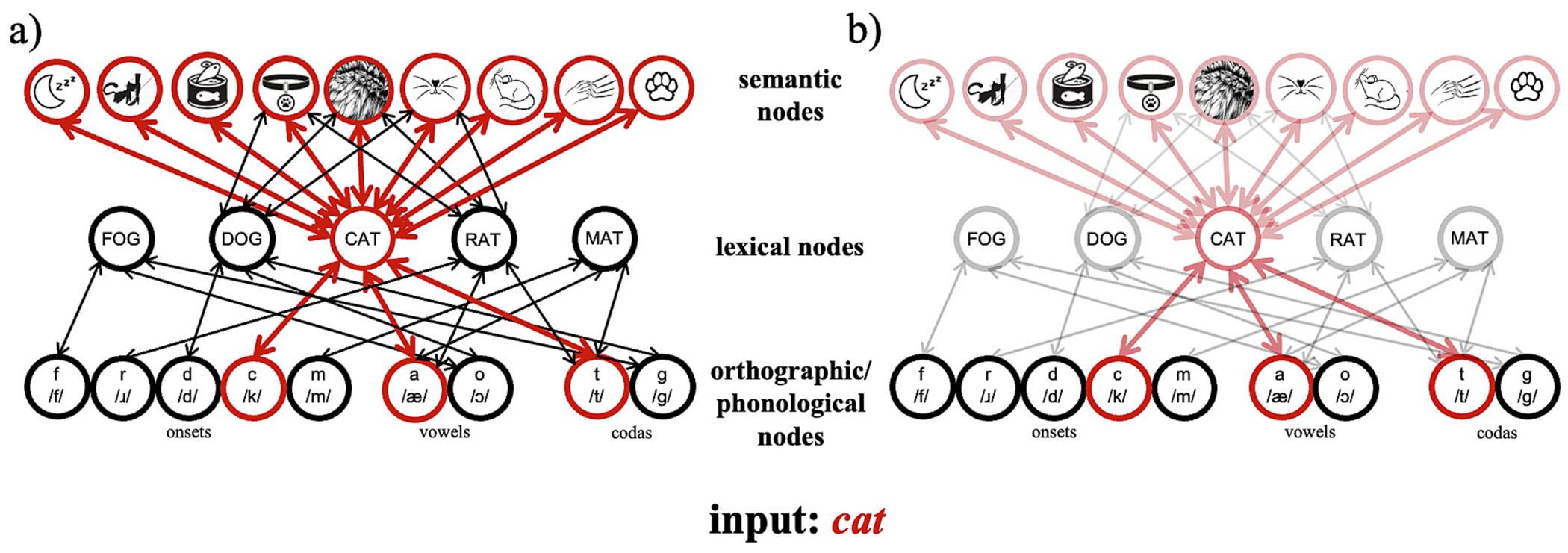
Models (adapted from Dell et al., 1997) of automatic spreading activation in (a) a typical system and (b) a system with an activation transmission deficit for incoming (auditory/orthographic) information. Activated nodes and connections are represented in shades of red. Faded lines represent weak connections, which reduce the spread of activation to other levels of the system, resulting in reduced activation of the nodes at those levels.

**Fig. 2.**
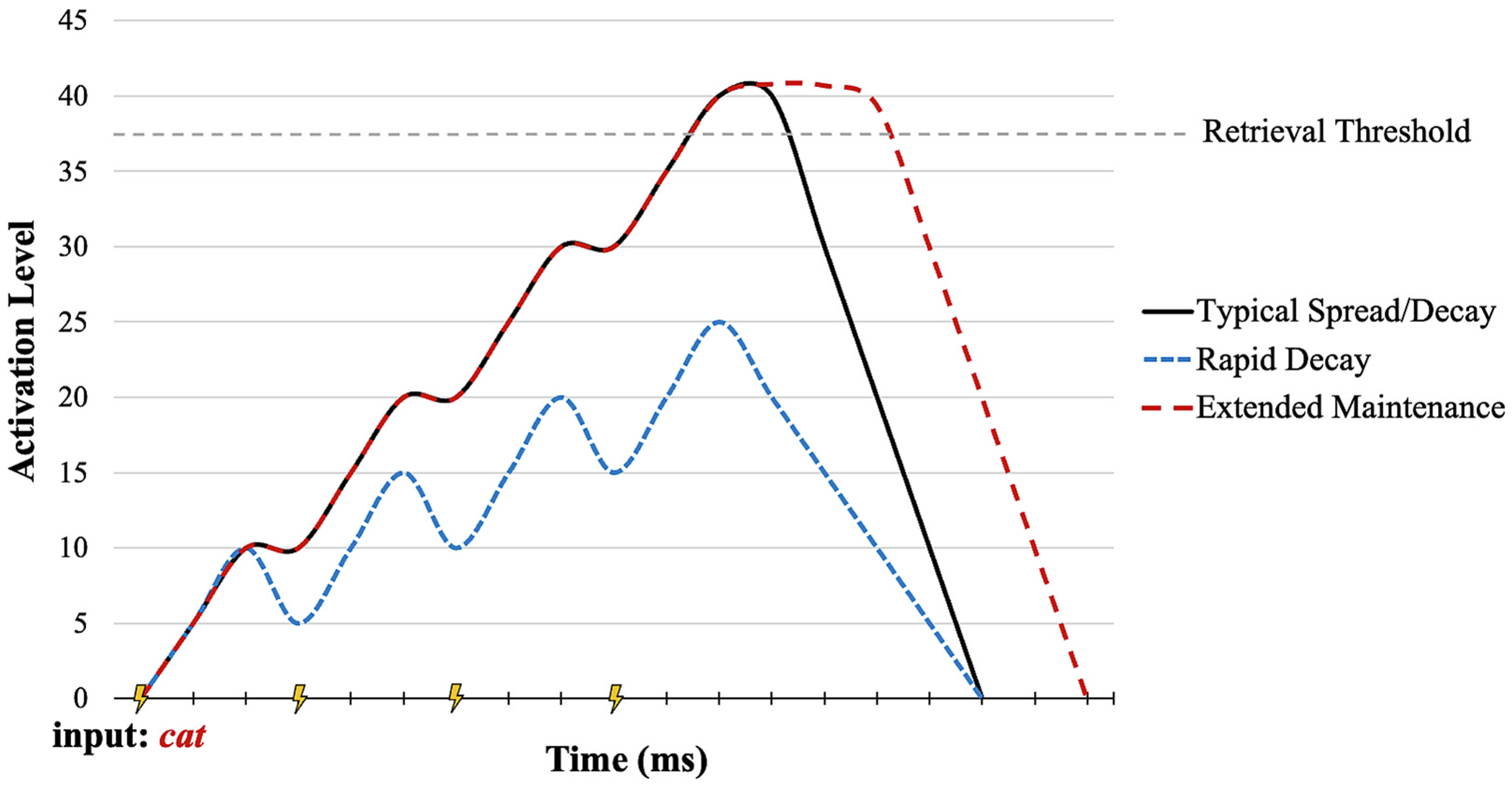
Depictions of a typical time course of spreading activation (black) and two different deficits in representation integrity: too-rapid decay of activation (dashed blue) and extended activation maintenance (dashed red). Lightning bolts symbolize “jolts” of activation over time from the bidirectional and interactive spread of activation across the levels of the system, which should allow target word activation to build until it reaches its retrieval threshold (i.e., for selection or recognition). With rapid activation decay, activation levels cannot build adequately, causing retrieval/recognition failure. With extended maintenance, activation levels do not decay in a timely manner, potentially creating processing interference.

Alterations in the speed of activation transmission or decay may underlie language deficits observed in aphasia, such as anomia. Indeed, anomia has been modeled (Dell et al., 1997) as resulting from either a deficit in *activation transmission*, in which activation fails to spread successfully within the lexical network (see Fig. 1), or a deficit in *representation integrity*, in which activation decays too rapidly or remains active too long to support accurate retrieval and downstream processing (see Fig. 2). This framework continues to guide the design and implementation of aphasia treatments (e.g., Boyle et al., 2018; Castro et al., 2022; Kalinyak-Fliszar et al., 2011).

### 1.2. Masked priming as a tool to understand implicit processing in aphasia

Additional evidence for impaired automatic spreading activation in aphasia comes from priming studies, in which exposure to one stimulus (i.e., the *prime*) facilitates processing of a subsequent stimulus (i.e., the *target*). One proposed mechanism underlying priming is automatic spreading activation: the prime’s activation spreads and increases the target’s activation, consequently easing access to the target (de Groot, 1983; Fowler et al., 1981; Neely, 1977).

Early priming studies in aphasia often used experimental paradigms that allowed both implicit and explicit processing to influence performance, such as list priming (e.g., Prather et al., 1992, 1997). Because participants consciously perceived all stimuli in these tasks, it is unclear whether the observed priming effects resulted from solely implicit processing (i.e., automatic spreading activation) and/or explicit processing (e.g., intentional use of strategies capitalizing on the prime-target relationship; e.g., Posner & Snyder, 1975; Silkes & Rogers, 2012).

Masked priming, by contrast, prevents the prime from reaching conscious awareness while still allowing it to influence processing (see Forster et al., 2003; Van den Bussche et al., 2009). Masking the primes (e.g., presenting interfering visual stimuli immediately before and/or after the briefly displayed prime) minimizes opportunities for explicit strategic or compensatory processing, isolating priming to automatic spreading activation. As a result, any observed effects can more confidently be attributed to implicit mechanisms, such as automatic spreading activation (Marcel, 1983; Van den Bussche et al., 2009).

Though limited, masked priming has been used with PWA, both to examine the time course of automatic spreading activation (Silkes & Rogers, 2012) and in treatment contexts targeting word retrieval impairments (Silkes, 2015, 2018; Silkes et al., 2013). The former line of study has provided valuable insight into the potential alterations in the time course of automatic spreading activation in aphasia. For example, using behavioral priming measures in a masked priming task, Silkes and Rogers (2012) found that PWA exhibited a later onset of priming effects and showed effects over a narrower range of interstimulus intervals (ISIs) compared to adults with typical language. These findings suggest both delayed initial spread and abnormally rapid decay of activation, consistent with explanations that link anomia to altered temporal dynamics of automatic spreading activation.

These prior studies have primarily relied on behavioral responses, which only indirectly index automatic spreading activation. Such measures reflect not only implicit processing of the prime, but also the explicit processing involved in stimulus judgments and overt motor responses. More direct measures of neural responses to primes can provide clearer insight into the timing and alterations in automatic spreading activation in aphasia.

### 1.3. Event-related potentials for studying the time course of implicit language processes

One way to better investigate the time course of language processing in aphasia is ERPs. Because ERPs do not require a behavioral response, they offer a direct index of implicit cognitive processing uncontaminated by motor responses (Bentin, 1989; Brandeis & Lehmann, 1986; Ibanez et al., 2012). Numerous studies combining ERPs with masked priming with unimpaired individuals have shown that the N400 reflects semantic processing and can serve as a marker of automatic spreading activation (e.g., Kiefer, 2002; Kutas & Federmeier, 2011; Kutas & Hillyard, 1980; Li et al., 2019; Liu et al., 2013; Misra & Holcomb, 2003). N400 priming effects typically present as attenuated amplitude to primed stimuli, reflecting facilitated processing (e.g., Chow et al., 2014; Rugg, 1985, 1990; Rugg & Nagy, 1989; Wilding et al., 1995). Critically, this attenuation occurs even when the prime is masked and not consciously perceived (e.g., Kiefer, 2002; Li et al., 2019; Liu et al., 2013; Misra & Holcomb, 2003), though often to a lesser extent than when primes are clearly visible (e.g., Brown & Hagoort, 1993; Holcomb et al., 2005; Misra & Holcomb, 2003). Thus, N400 priming effects reflect the implicit, automatic, lexical-semantic processing driven by spreading activation.

Misra and Holcomb (2003) provided especially compelling evidence for this interpretation through a combined masked and visible repetition priming paradigm. Participants performed a go/no-go task, responding only to animal names, while ERP responses to prime-target word pairs were recorded. Targets were either unprimed or repetitions of primes (i. e., primed), occurring either immediately (i.e., within the same trial) or after a delay (i.e., in a later trial). ERP responses to non-animal targets revealed N400 attenuation (i.e., priming effects) for repetitions of both masked and visible primes, but only repetitions of visible primes elicited a late positive component (LPC)—a component associated with conscious perception and subsequent recognition of stimuli (Düzel et al., 1997; Rugg, 1990; Rugg & Nagy, 1989; Schnyer et al., 1997; van Petten et al., 1991). This dissociation demonstrates that the N400 priming effects for repetitions of masked primes reflect solely implicit, automatic spreading activation rather than residual awareness of visible primes or other explicit mechanisms. Later studies have also corroborated that masked N400 priming effects arise from automatic spreading activation (e.g., Kiefer, 2002; Liu et al., 2013).

Importantly, Misra and Holcomb’s (2003) paradigm also enabled investigation of the time course of automatic spreading activation. By varying prime-target ISIs from less than 500 ms (i.e., immediate, within-trial repetitions) to over 25000 ms (i.e., up to eight trials later), a graded pattern of masked N400 priming effects appeared: attenuation was greatest for immediate repetitions and smaller, but still present, for delayed repetitions. This pattern illustrates the typical time course of spreading activation as indexed by masked N400 priming effects and establishes the masked priming ERP paradigm with varied prime-target ISIs as optimal for examining the temporal dynamics of automatic spreading activation. Misra and Holcomb’s (2003) paradigm is therefore well suited for exploring the time course of spreading activation in other populations, such as PWA.

### 1.4. Present study

This study extended Misra and Holcomb’s (2003) masked priming ERP paradigm to PWA. Both masked priming and ERPs have been validated for use with this population (see Silkes & Anjum, 2021 for a review of the use of ERPs with PWA). The task’s low linguistic demands and absence of a verbal response requirement make it feasible for PWA, and its use of masked priming specifically minimizes the likelihood of strategic processing or intentional application of compensatory strategies, allowing isolation of implicit processes. Including both masked and visible primes, along with varied ISIs, allowed us to examine priming effects over time.

The primary aim of this study was to investigate the time course of spreading activation in PWA relative to age-matched adults with typical language. Within each group, we compared ERP responses to immediate and delayed repetitions of masked and visible primes. Based on prior research, we predicted that typical adults would show N400 priming effects for immediate repetitions of masked primes and delayed repetitions of visible primes, but minimal or no effects for delayed repetitions of masked primes. In contrast, and central to our investigation, we predicted that PWA would show delayed initial spread of activation, demonstrating no N400 priming effects for immediate repetitions of masked primes but reliable N400 priming effects for delayed repetitions of masked and visible primes.

While we primarily focused on N400 priming effects, we also analyzed behavioral responses and two additional ERP components to validate the experimental design. Behaviorally, we examined probe identification accuracy during the priming task, behavioral priming effects for non-critical probe words, and recognition of critical words in a post-hoc recognition task. We predicted that both groups would show behavioral priming effects (i.e., faster responses to primed than unprimed probe targets; e.g., Ferrill et al., 2012; Prather, 1994; Prather et al., 1992, 1997; Silkes, 2012; Silkes et al., 2020). The remainder of the behavior analyses were conducted to solely confirm that the masked prime words were not consciously recognized.

We also analyzed the P300 elicited by probe words to assess conscious detection of task-relevant stimuli, and analyzed the LPC elicited by all critical target types to assess for perception of masked versus visible primes. We predicted these components would confirm that the *masked* words were neither reliably identified nor consciously processed, while the *visible* words were. Behavioral priming and probe identification results are reported here; however, given the supplementary nature of these analyses, full methods and results are presented in the Supplemental Materials. See Table 1 for a detailed summary of all the ERP predictions.

## 2. Method

Participants were recruited through the University of Washington Aphasia Registry and Repository and via advertisements posted on the University of Washington campus. All procedures were approved by the University of Washington Institutional Review Board. Informed consent was obtained from all participants prior to participation, using multimodal communication with PWA to ensure their comprehension.

Following consent, participants were screened for eligibility using the Boston Naming Test (BNT; Kaplan et al., 2001), Raven’s Coloured Progressive Matrices (RCPM; Raven, 1976), and a vision assessment using a Tumbling E eye chart. PWA also completed the Western Aphasia Battery (WAB; Kertesz et al., 1982) to establish an aphasia diagnosis and profile. Eligible participants then completed a task to determine the appropriate duration for the masked primes (see Sections 2.2.1 and 2.3.1), defined as the longest of three durations at which participants’ performance responding to masked words was near chance. Participants who met all eligibility criteria proceeded to complete the experimental protocol, which included EEG and behavioral data collection during a priming task. Participants completed all tasks in up to two sessions and received nominal monetary compensation for their participation, prorated based on the extent of their involvement.

### 2.1. Participants

A total of 51 participants were screened for participation in this study (PWA: *n* = 22; Controls: *n* = 29), but the full experimental protocol was not completed by two PWA due to visual field impairments (*n* = 1) or inability to control their eyeblinks (*n* = 1) and by 10 Controls due to inability to obtain adequate prime duration thresholds (i.e., they showed above-chance conscious perception of primes at even the fastest duration; details in Section 2.3.1). Of the 39 participants that completed the full experimental protocol, data collected from 12 participants (PWA: *n* = 6; Controls: *n* = 6) were excluded due to excessive EEG artifacts (see Section 2.5 for more details). Thus, data from a total of 27 right-handdominant, monolingual, English-speaking adults (PWA: *n* = 14; Controls: *n* = 13) were included in this study. Participants had no reported history of neurological injury or impairment (other than the event that caused aphasia for the PWA) or untreated psychiatric conditions and had normal or corrected-to-normal vision.

All PWA had experienced a left-hemisphere stroke at least six months prior to the study (*M* = 5.66 years, *SD* = 3.76) and been diagnosed with aphasia. All had an Aphasia Quotient (AQ) between 22.8 and 94.7/100 (*M* = 68.01, SD = 21.83) on the WAB, indicating the presence of impaired language, and scored below 48/60 (*M* = 27.93, *SD* = 17.33) on the BNT, confirming anomia. None showed evidence of right hemisphere impairment (all scores ≥ 23/36; *M* = 31.36, *SD* = 4.03) on RCPM or of visual field cut or neglect. PWA ranged from 33 to 74 years old (*M* = 60.36, *SD* = 11.34). See Table 2 for individual participant characteristics.

All control participants scored at least 52/60 on the BNT and at least 23/36 on the RCPM, indicating no left- or right-hemisphere damage, respectively. Control participants ranged from 45 to 74 years old (*M* = 58.69, *SD* = 9.52; not significantly different from the PWA, *t*(24.77) = 0.41, *p* = 0.682).

### 2.2. Stimuli

Each participant completed two priming tasks: one to determine the appropriate prime presentation duration and the main experimental task. Stimuli consisted of the same words and masking sequences used by Misra and Holcomb (2003).

#### 2.2.1. Prime duration determination task

To account for individual variability in sensitivity to masking (Cheesman & Merikle, 1984; Dagenbach et al., 1989; Misra & Holcomb, 2003), participants completed a task to determine the masked prime duration for the experimental task that ensured effective masking for each individual (i.e., primes were masked enough that they were unable to identify the prime’s semantic category). In this task (described more fully in Section 2.3.1), participants pressed a button when they saw the name of something to eat or drink. Stimuli for this task consisted of 257 four- to five-letter words, 105 of which were names of food items (probes) and 152 of which were non-food, concrete nouns. Stimuli were divided into three lists (one per tested prime duration), each with a total of 51 trials. Probes appeared as both masked primes and visible targets, but never both within the same trial. Some trials presented a masked prime probe, some a visible target probe, and others only non-food items.

#### 2.2.2. Experimental priming task

The experimental priming task (described more fully in Section 2.3.2) involved viewing masked and visible words and pressing a button whenever the name of an animal appeared. Stimuli for the 450 experimental trials consisted of 500 four- to five-letter words with a frequency of less than 121.6 per million (*M* = 8.14 per million, *SD* = 10.98 per million; Brysbaert & New, 2009), including 100 animal names (probes) and 400 non-animal, concrete nouns (critical words). Fifty of the critical words were never presented in the experimental priming task to serve as foils in the recognition task (see Supplemental Materials).

Stimuli were divided into eight counterbalanced stimulus lists, each with 12 blocks of 36 trials and one block of 18 trials. Animal-name probes appeared as masked primes and visible targets, but never as both within a single trial. As masked primes, they indexed whether participants consciously perceived the masked words; as visible targets, they promoted task engagement and were intended to elicit behavioral masked priming effects. Non-animal critical words appeared in both prime and target positions and were sometimes repeated within a trial. Words in the prime position were always masked primes. Words in the target position were always visible targets and also served as visible primes in some trials (i.e., appeared twice in the target position, with no word in the prime position on either trial). Including both masked and visible primes encouraged continuous attention to the prime epoch regardless of prime perceptibility, increasing the likelihood of eliciting masked priming effects, as masked primes alone may not reliably do so (Holcomb et al., 2005; Kahan, 2000).

This design yielded ten stimulus types, illustrated in a representative stimulus list in Table 3. Of these, five were critical stimulus types used to elicit the neural priming effects. Two were unprimed targets:

1. No-prime targets (i.e., unprimed targets with a blank screen in the prime position);
2. Unrelated-prime targets (i.e., unprimed targets with an unrelated word in the prime position).
3. The remaining three were primed targets:
4. Delayed repetitions of visible primes;
5. Immediate repetitions of masked primes;
6. Delayed repetitions of masked primes.

Trials with immediate repetitions of masked primes always had a word in the prime position, whereas delayed repetitions of primes always had a blank screen in the prime position. To create identical trial structures when comparing unprimed and primed targets, some unprimed trials had a masked prime unrelated to the target word in the prime position and other unprimed trials had a blank screen in the prime position. This manipulation enabled comparison of all primed critical target types to a baseline target with no potential for either facilitated processing through priming or inhibited processing through anti-priming (e.g., Ramanathan, 2009).

In addition to the five critical stimulus types, there were five additional stimulus types, most of which served as probes:

1. Masked primes (i.e., the non-animal masked primes for stimulus types #4 and #5);
2. Masked prime probes (i.e., the masked animal primes for stimulus type #9; probe);
3. Unprimed probe targets (i.e., animal targets with a blank screen in the prime position; probe);
4. Primed probe targets (i.e., delayed repetitions of masked animal primes; probe);
5. Filler targets (i.e., non-animal words that immediately followed masked animal prime probes; not analyzed).

### 2.3. Procedure

**Fig. 3.**
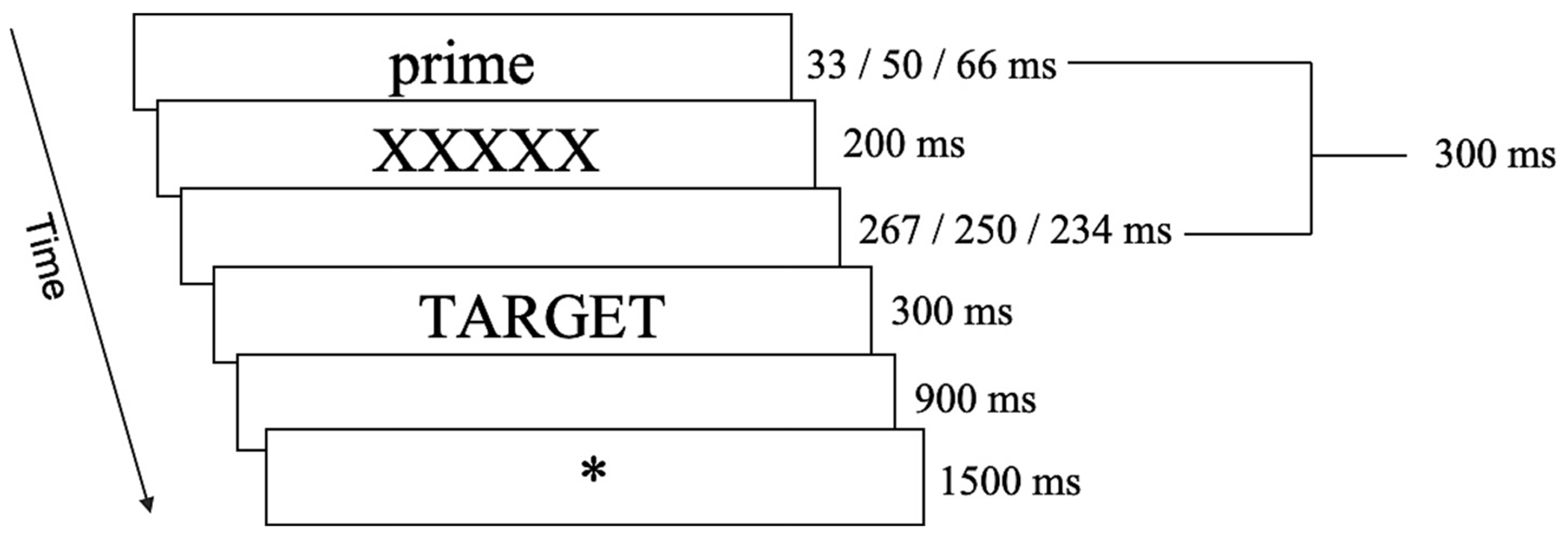
Stimulus presentation sequence for a single trial. Primes were presented for 33, 50, or 66 ms (see text for details), with the blank screen following the prime adjusted so that the combined duration of the two screens was 300 ms. This, combined with the backward mask duration, created a 500 ms SOA between the prime and target.

Participants completed the prime duration determination task before completing the experimental priming task. In both tasks, each trial involved sequentially presenting masked and visible words, as shown in Fig. 3, presented in 30-point Arial font via E-Prime in the middle of a 20″ ViewSonic P225f CRT monitor connected to a desktop PC running Windows XP. Participants completed the tasks seated approximately 17 in. from a computer monitor in a comfortable office chair in a sound-attenuated and dimly lit room. They were told they would see items flashed on the screen, including words, strings of X’s, and asterisks, and were instructed to watch carefully and respond as quickly as possible by pressing a button on an E-Prime response box with color-coded keys whenever they saw a word from the designated category (food for the prime duration determination task; animals for the experimental priming task). This category-specific response requirement ensured participants semantically processed each word and prevented movement-related electrical activity generated by the button presses from contaminating the EEG data for critical words (i.e., non-food or nonanimal words). Participants were not informed of the presence of masked items or the prime-target relationship. To facilitate engagement, the tasks included frequent opportunities for breaks, which participants could take for as long as needed. Wakefulness was monitored throughout both tasks via real-time visual inspection of the EEG signal. If substantial alpha activity was detected, researchers intervened via intercom and/or offered a snack and/or water during a break. Participants were also continuously supervised through a one-way mirror for the duration of the tasks.

#### 2.3.1. Prime duration determination task

To determine the appropriate prime duration for each participant, they completed three sets of trials, referred to as “practice runs,” each with a different prime duration: 66 ms, 50 ms, and 33 ms, in that order. Each trial included either a blank screen or a masked word unrelated to the target word in the prime position, followed by a visible target word. Blocks within each set were separated by breaks, during which the experimenter provided supportive and/or clarifying feedback.

After completing all three sets, the experimenter calculated the percentage of probes in the prime position correctly responded to, using the E-Prime response data after correcting for self-reported button errors (e.g., immediately following a button press with, “Whoops, I meant to hit the other button”). Following Misra and Holcomb’s (2003) criteria, the longest duration at which the participant achieved between 33 and 66% accuracy (with as close to 50% accuracy as possible) was selected as the participant’s masked prime duration for the experimental priming task.

#### 2.3.2. Experimental priming task

Trials in the experimental priming task (see Fig. 3) began with a prime stimulus presented for the duration established for that participant (33 ms: 11 Controls, 4 PWA; 50 ms: 2 Controls, 4 PWA; 66 ms: 0 Controls, 6 PWA). This was followed by a backward visual mask presented for 200 ms, and then a blank screen presented for the duration necessary to create a 500 ms stimulus onset asynchrony (SOA) between the prime and target (e.g., 267 ms blank screens for 33 ms primes; 234 ms blank screens for 66 ms primes). The target word then appeared for 300 ms, followed by a blank screen for 900 ms, and then an asterisk for 1500 ms, which signaled the end of the trial and that blinking was permitted.

Participants were told that the experimental priming task was identical to the earlier “practice” task, except that they should now respond to animal names. Movement artifacts were briefly explained, and participants were asked to try to remain relaxed and limit blinking to the post-trial interval marked by the asterisk. To familiarize participants with the timing for eyeblinks and the new response instructions, they completed a set of 51 practice trials using their predetermined prime duration. They were permitted to repeat the full set of practice trials as many times as desired before starting the experimental task. The entire experimental priming task lasted approximately 25 min, excluding breaks between lists.

### 2.4. EEG procedure

EEG data were recorded continuously at 2048 Hz using a BioSemi ActiveTwo EEG system (BioSemi B.V., Amsterdam, The Netherlands) with active Ag-AgCl electrodes mounted on a 64-channel elastic cap (Electro-Cap, Inc.) organized following the extended International 10–20 system. The system’s active electrode design allows the preamplifier stage to compensate for high electrode impedances to maintain a good signal-to-noise ratio; therefore, impedance values were not recorded but were monitored during electrode placement. Additional monopolar electrodes were placed below the left eye and lateral to the right eye to monitor eyeblinks and horizontal eye movements, respectively. Another monopolar electrode was placed on the left mastoid for offline re-referencing from the system’s online common mode sense (CMS) reference (positioned near Pz; Metting van Rijn et al., 1990).

### 2.5. EEG data processing

EEG data were processed in MATLAB using the open-source toolboxes EEGLAB (version 2021.0; Delorme & Makeig, 2004) and ERPLAB (version 8.10; Lopez-Calderon & Luck, 2014). Initial data (pre)processing steps included: 1) downsampling to 500 Hz; 2) bandpass filtering using a second-order Butterworth filter with half-amplitude bandpass of 0.1–30 Hz; 3) re-referencing to the left mastoid; 4) visually identifying and removing data from malfunctioning electrodes; 5) decomposing the data via independent component analysis (ICA); and 6) removing ICA components associated with ocular and/or muscular movement artifacts (range = 0–3 components, *M* = 1.8 components). Data from each participant were then segmented into epochs extending 100 ms before target onset (i.e., baseline period) to 1180 ms post-onset (i.e., trial end). Epochs containing horizontal eye movements, blinks, and/or non-neural voltage deflections exceeding 75 μV were rejected. Data from participants with more than 50% of epochs rejected were excluded from the analyses. On average, 81.42% of epochs (*SD* = 11.92%) were retained for Controls and 75.19% (*SD* = 14.54%) for PWA. There were no significant differences in the proportion of trials rejected across stimulus types or between groups (all *p*s *>* 0.05).

### 2.6. Data analysis

Behavioral and EEG data were analyzed with linear mixed effects (LME) regression models fit via restricted maximum likelihood (REML) estimation, implemented with the *lme4* package (version 1.1–35.1; Bates et al., 2015) in R (version 4.3.2; R Core Team, 2023). Bonferroni correction was applied to control for planned multiple comparisons of conditions (not across all points within a time window or model estimates; see Table 1), maintaining a family-wise type I error rate of 0.05. These comparisons were *a priori* contrasts tested within the model, ensuring that only a limited set of comparisons were subject to correction (see Schad et al., 2020 for the details of this approach).

Arcsine-transformed proportions of correct button-press responses to animal names were analyzed with an LME model that included fixed within-subject factors of Stimulus Type (Prime, Primed Target, Unprimed Target) and Group (PWA, Controls), with a random intercept for participant. The model was run with both treatment and Helmert coding to compare: 1) response accuracy for each type of animal target relative to animal primes (treatment coding with animal primes as the reference level); 2) primed versus unprimed animal targets; and 3) animal primes versus all animal targets (both primed and unprimed; Helmert coding), separately for each group.

To analyze the behavioral priming effects, we ran an LME model on reaction times to correctly identified animal targets. The model included fixed within-subject factors of Priming (Primed, Unprimed) and Group (PWA, Controls), with random intercepts for participant and item. Treatment coding was applied, making the intercept reflect the Controls’ mean reaction time for unprimed trials. Trials with reaction times ±3 SDs from a participant’s mean reaction time were excluded from probe detection and behavioral priming effect analyses (trials removed: Controls: *M* = 15.40%, *SD* = 8.20%; PWA: *M* = 10.01%, *SD* = 11.68%).

N400 ERP priming effects were analyzed by extracting mean amplitudes for each participant between 300 and 500 ms post-target onset from 21 electrodes: F3, Fz, F4, FC3, FCz, FC4, C3, Cz, C4, CP3, CPz, CP4, P3, Pz, P4, PO3, POz, PO4, O1, Oz, and O2. The time window and electrodes were selected based on prior masked N400 priming studies with neurologically unimpaired adults (e.g., Kiefer, 2002; Kiefer & Brendel, 2006; Misra & Holcomb, 2003; Ortells et al., 2016) and N400 priming studies with PWA (e.g., Chang et al., 2016). The amplitudes were analyzed using an LME model with fixed within-subject factors of Target Type (No-Prime Target, Delayed Repetition of Masked Prime, Delayed Repetition of Visible Prime, Immediate Repetition of Masked Prime, Unrelated-Prime Target) and Group (PWA, Controls), and random intercepts for participant and electrode. We ran the model using both treatment and repeated contrast coding to test the planned comparisons of target types for each group.

After identifying the target types that elicited priming effects, we conducted additional analyses to compare the magnitudes of the priming effects. We calculated mean amplitudes of difference waves by subtracting the mean amplitude of the primed condition from the mean amplitude of the unprimed condition for each electrode and participant. The resulting amplitudes were analyzed using an LME model with fixed within-subject factors of Difference Condition (No-Prime Targets – Delayed Repetitions of Visible Primes, No-Prime Targets – Delayed Repetitions of Masked Primes, No-Prime Targets – Immediate Repetitions of Masked Primes, Unrelated-Prime Targets – Immediate Repetitions of Masked Primes) and Group (PWA, Controls), with random intercepts for participant and electrode. Treatment coding was applied with the No-Prime Targets – Delayed Repetitions of Masked Primes Difference Condition as the reference level, so the intercept represented the grand mean amplitude for that condition in Controls.

## 3. Results

### 3.1. Behavioral results

#### 3.1.1. Animal name probe identification: Verifying masking effectiveness and task engagement

Button press responses to animal name probes revealed that both Controls and PWA identified a significantly greater proportion of visible probe targets, regardless of whether they were primed or unprimed, than masked prime probes (Controls: Primes vs. Primed Targets: *β* = 2.52, *SE* = 0.26, *t*(73) = 9.51, *p* < 0.001; Primes vs. Unprimed Targets: *β* = 2.61, *SE* = 0.26, *t*(73) = 9.87, *p* < 0.001; PWA: Primes vs. Primed Targets: *β* = 2.53, *SE* = 0.26, *t*(73) = 9.92, *p* < 0.001; Primes vs. Unprimed Targets: *β* = 2.26, *SE* = 0.26, *t*(73) = 8.88, *p* < 0.001). However, there was no significant difference in response accuracy between primed and unprimed probe targets (Controls: *β* = −0.05 *SE* = 0.13, *t*(73) = −0.37, *p* = 0.716; PWA: *β* = 0.13, *SE* = 0.13, *t*(73) = 1.04, *p* = 0.302). Response accuracy by probe type is summarized in Table 4.

#### 3.1.2. Reaction times: Examining behavioral priming effects

To examine behavioral priming effects, reaction times to primed and unprimed probe targets were analyzed. Neither group showed behavioral priming effects, as reaction times did not significantly differ between primed and unprimed probe targets (Controls: *β* = −21.85, *SE* = 20.61, *t*(1305) = −1.06, *p* = 0.289; PWA: *β* = −15.34, *SE* = 15.95, *t*(1305) = −0.96, *p* = 0.336). However, PWA responded more slowly overall (*M* = 1446 ms, *SD* = 281 ms) than Controls (*M* = 1312 ms, *SD* = 215 ms; *β* = 165.00, *SE* = 21.31, *t*(1305) = 7.74, *p* < 0.001).

### 3.2. ERP results

#### 3.2.1. Delayed repetitions of visible primes versus unprimed targets: Verifying elicitation of neural priming effects reflecting implicit and explicit processing

**Fig. 4.**
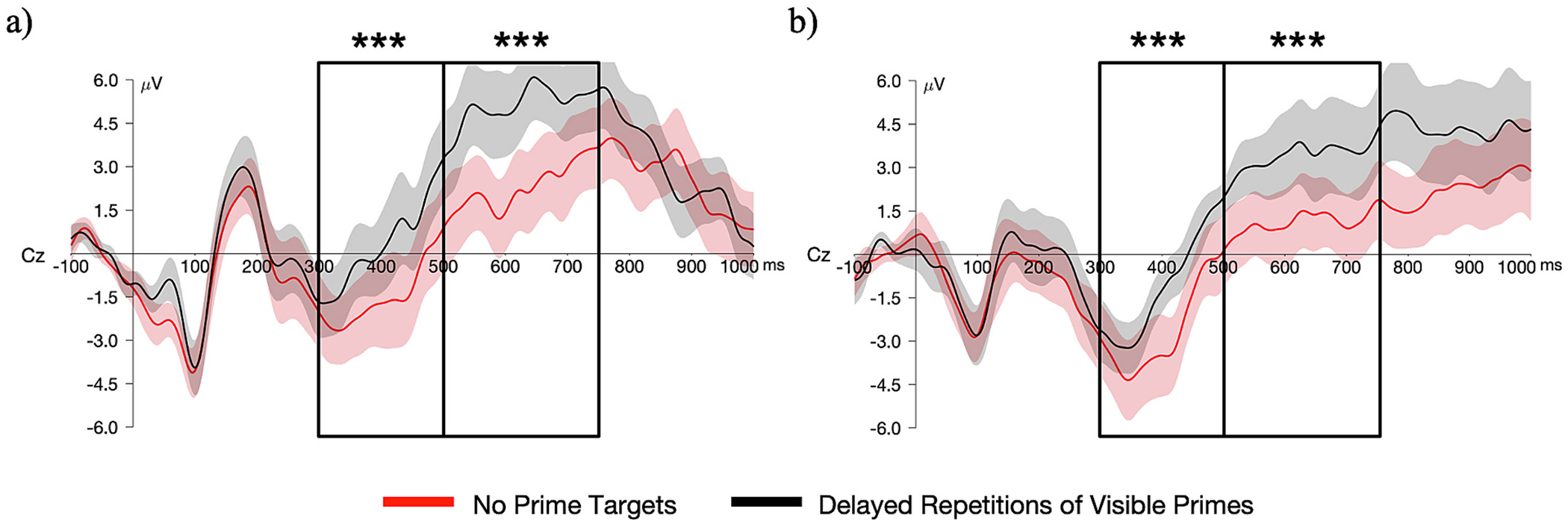
Grand average ERP waveforms at electrode Cz for a) Controls and b) PWA elicited by delayed repetitions of visible primes (black) and no-prime targets (red). Shaded regions indicate ± 1 standard error of the mean. Vertical boxes highlight the analyzed N400 and LPC time windows. Statistical significance is denoted by asterisks (*** *p* < 0.001).

To verify that priming effects could be elicited with the strongest form of priming, when both implicit and explicit processing are involved, we analyzed mean amplitudes for Delayed Repetitions of Visible Primes compared to No-Prime Targets (see Fig. 4). Both groups showed priming effects: Controls exhibited significantly reduced amplitudes for Delayed Repetitions of Visible Primes relative to No-Prime Targets (*β* = 6.63, *SE* = 1.02, *t*(2822) = 6.51, *p* < 0.001), as did PWA (*β* = 4.95, *SE* = 0.98, *t*(2822) = 5.04, *p* < 0.001).

#### 3.2.2. Immediate repetitions of masked primes versus unprimed targets: Examining automatic priming effects and speed of initial spread of activation

**Fig. 5.**
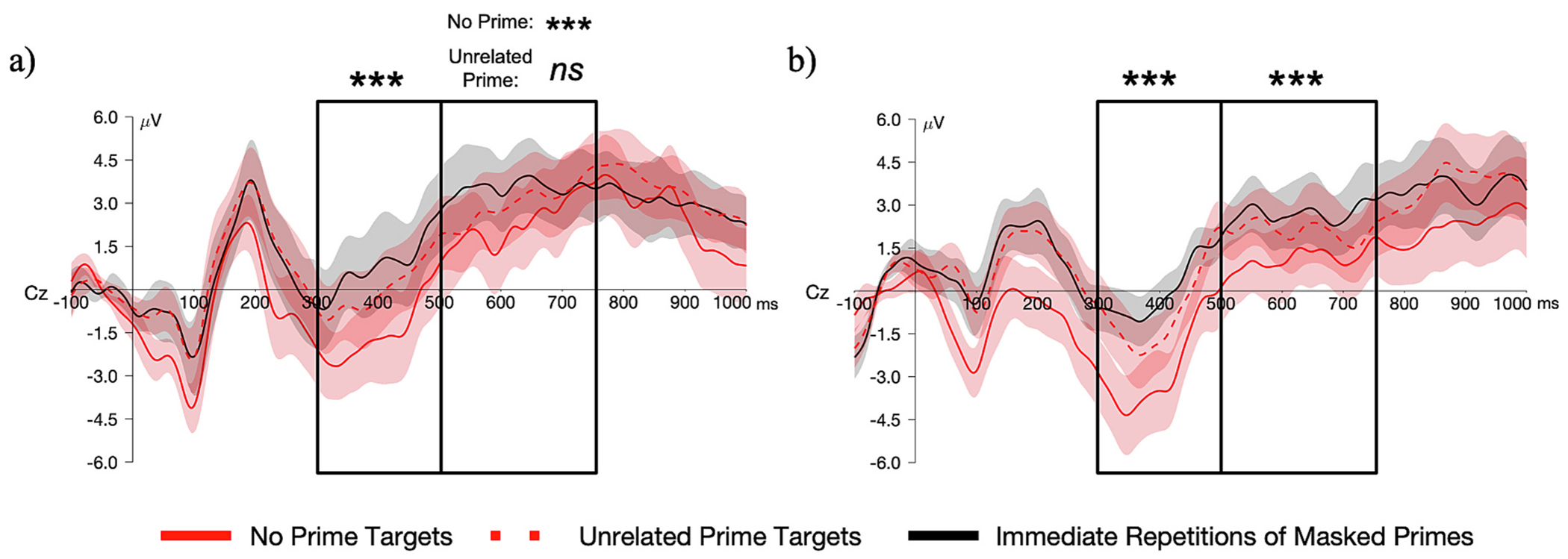
Grand average ERP waveforms at electrode Cz for a) Controls and b) PWA elicited by immediate repetitions of masked primes (black), no-prime targets (solid red), and unrelated-prime targets (dashed red). Shaded regions indicate ± 1 standard error of the mean. Vertical boxes highlight the analyzed N400 and LPC time windows. Statistical significance is denoted by asterisks (*** *p* ≤ 0.001; *ns* = not significant).

To address our main hypothesis and determine whether PWA show a typical or altered ability to rapidly spread activation in response to a stimulus, we compared mean amplitudes for Immediate Repetitions of Masked Primes to both the No-Prime Targets and Unrelated-Prime Targets (see Fig. 5). Both Controls and PWA showed priming effects for Immediate Repetitions of Masked Primes relative to each unprimed condition (Controls: No-Prime Targets: *β* = 9.51, *SE* = 1.02, *t*(2822) = 9.33, *p* < 0.001; Unrelated-Prime Targets: *β* = −3.48, *SE* = 1.02, *t*(2822) = −3.41, *p* = 0.001; PWA: No-Prime Targets: *β* = 10.35, *SE* = 0.97, *t* (2822) = 10.54, *p* < 0.001; Unrelated-Prime Targets: *β* = −4.25, *SE* = 0.97, *t*(2822) = −4.33, *p* < 0.001).

#### 3.2.3. Delayed repetitions of masked primes versus unprimed targets: Examining automatic priming effects and speed of spread and maintenance of activation

**Fig. 6.**
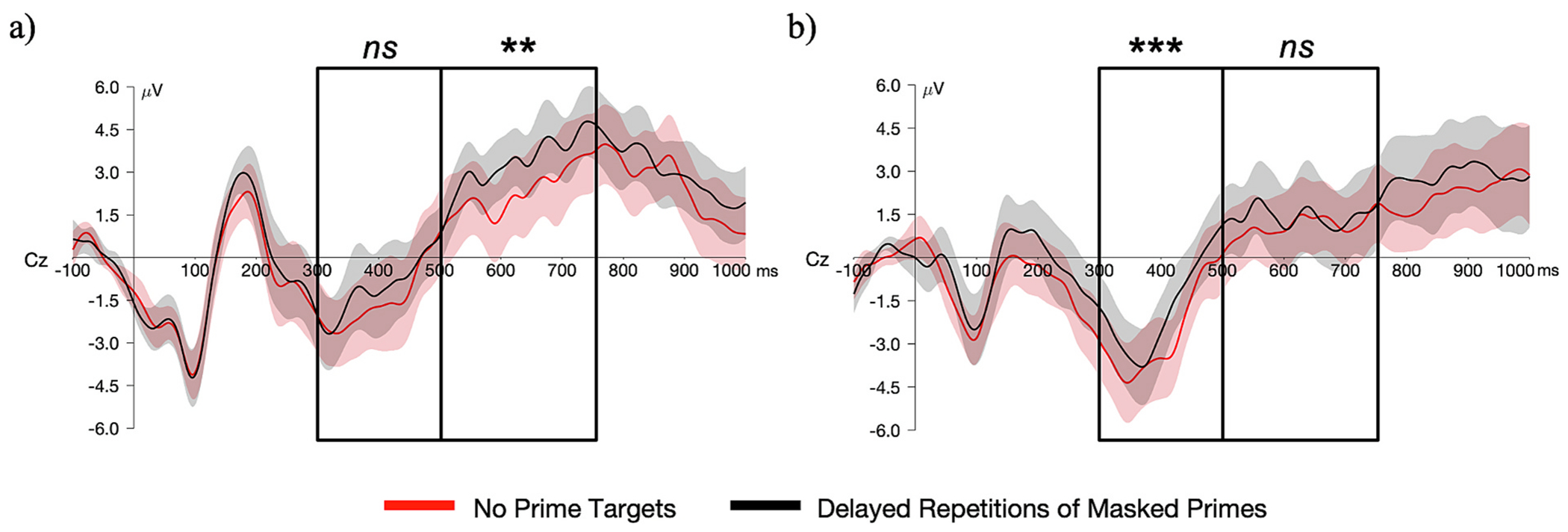
Grand average ERP waveforms at electrode Cz for a) Controls and b) PWA elicited by delayed repetitions of masked primes (black) and no-prime targets (red). Shaded regions indicate ± 1 standard error of the mean. Vertical boxes highlight the analyzed N400 and LPC time windows. Statistical significance is denoted by asterisks (*** *p* < 0.001, ** *p* < 0.01; *ns* = not significant).

To further compare the speed of initial spreading activation, as well as the maintenance of activation over time, between PWA and Controls, we analyzed mean amplitudes for Delayed Repetitions of Masked Primes relative to No-Prime Targets (see Fig. 6). Controls did not show any priming effects for Delayed Repetitions of Masked Primes (*β* = 1.75, *SE* = 1.02, *t*(2822) = 1.72, *p* = 0.086), but PWA did (*β* = 3.61, *SE* = 0.98, *t* (2822) = 3.68, *p* < 0.001).

#### 3.2.4. Immediate repetitions of masked primes versus delayed repetitions of masked primes: Further examining automatic priming effects and speed of spread and maintenance of activation

**Fig. 7.**
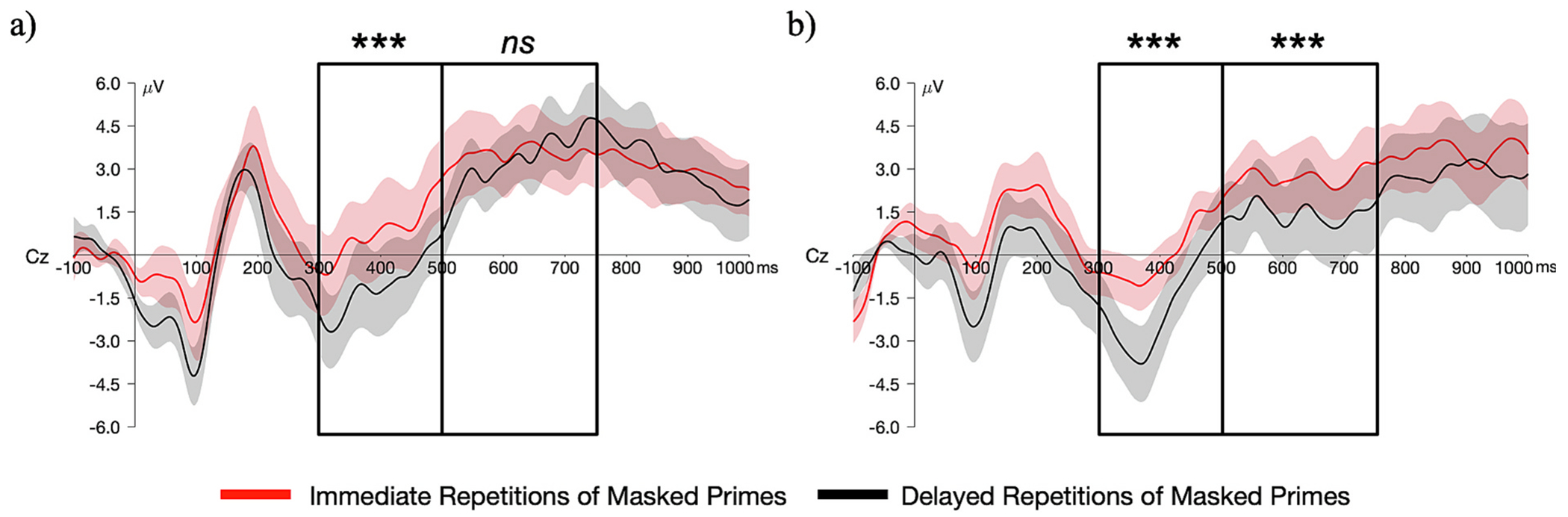
Grand average ERP waveforms at electrode Cz for a) Controls and b) PWA elicited by immediate repetitions of masked primes (red) and delayed repetitions of masked primes (black). Shaded regions indicate ± 1 standard error of the mean. Vertical boxes highlight the analyzed N400 and LPC time windows. Statistical significance is denoted by asterisks (*** *p* < 0.001; *ns* = not significant).

Because PWA showed priming effects for both Immediate and Delayed Repetitions of Masked Primes, we conducted exploratory analyses comparing mean amplitudes of these two target types. Both Controls and PWA showed significantly more negative mean amplitudes for Delayed Repetitions of Masked Primes compared to Immediate Repetitions of Masked Primes (Controls: *β* = 7.76, *SE* = 1.02, *t*(2822) = 7.61, *p* < 0.001; PWA: (*β* = 6.74, *SE* = 0.98, *t*(2822) = 6.86, *p* < 0.001; see Fig. 7).

**Fig. 8.**
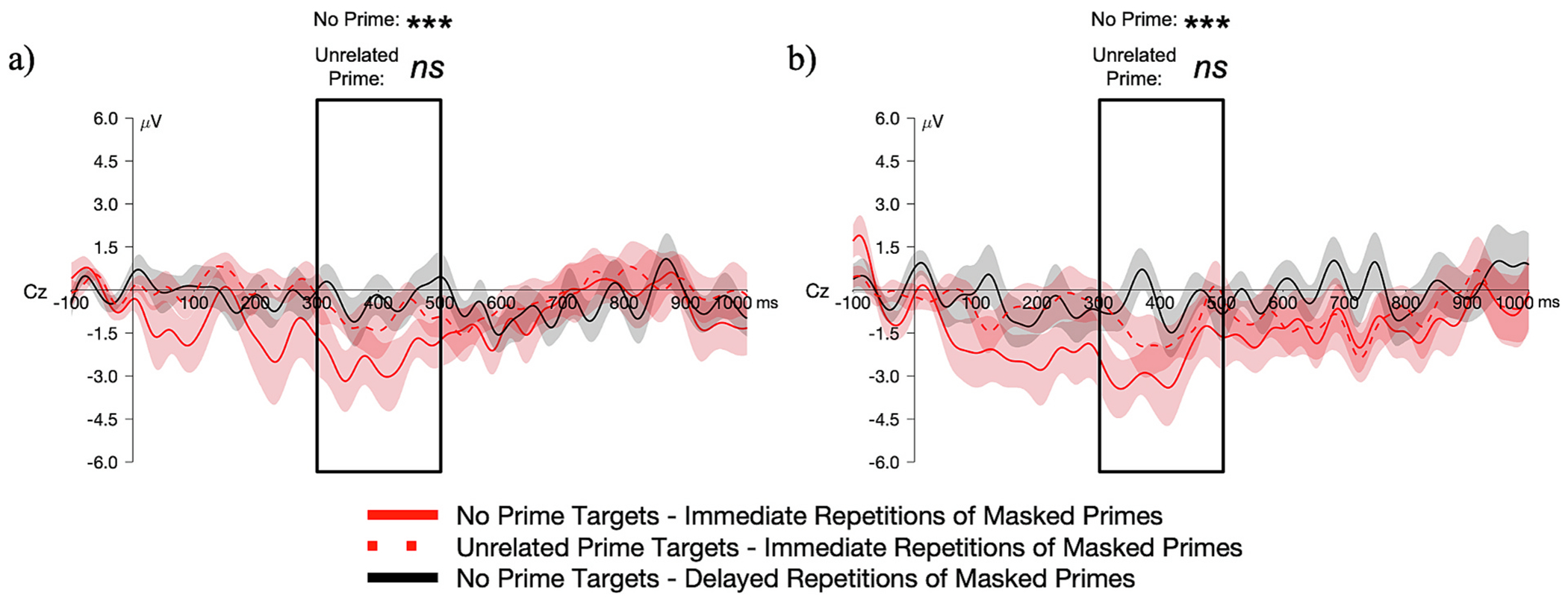
Grand average difference waves at electrode Cz for a) Controls and b) PWA, comparing immediate repetitions of masked primes (with two baseline conditions: no-prime targets in solid red and unrelated-prime targets in dashed red) and delayed repetitions of masked primes (black). More negative deflections indicate larger priming effects. Shaded regions indicate ± 1 standard error of the mean. Vertical boxes highlight the analyzed N400 time window. Statistical significance is denoted by asterisks (*** *p* < 0.001; *ns* = not significant).

To more closely examine these differences, we compared the magnitude of priming effects using mean amplitudes of difference waves (unprimed minus primed condition; see Fig. 8). When the No-Prime Targets served as the unprimed condition, both PWA and Controls showed greater priming effects for Immediate than Delayed Repetitions of Masked Primes (PWA: *β* = −6.74, *SE* = 0.86, *t*(2257) = −7.81, *p* < 0.001; Controls: *β* = −7.76, *SE* = 0.90, *t*(2257) = −8.67, *p* < 0.001). However, this difference was not significant when Unrelated-Prime Targets were used as the unprimed condition (PWA: *β* = −0.64, *SE* = 0.86, *t*(2257) = −0.74, *p* = 0.457; Controls: *β* = −1.72, *SE* = 0.90, *t*(2257) = −1.93, *p* = 0.054).

#### 3.2.5. Delayed repetitions of masked primes versus delayed repetitions of visible primes: Examining priming effects reflecting only implicit processing or implicit and explicit processing

**Fig. 9.**
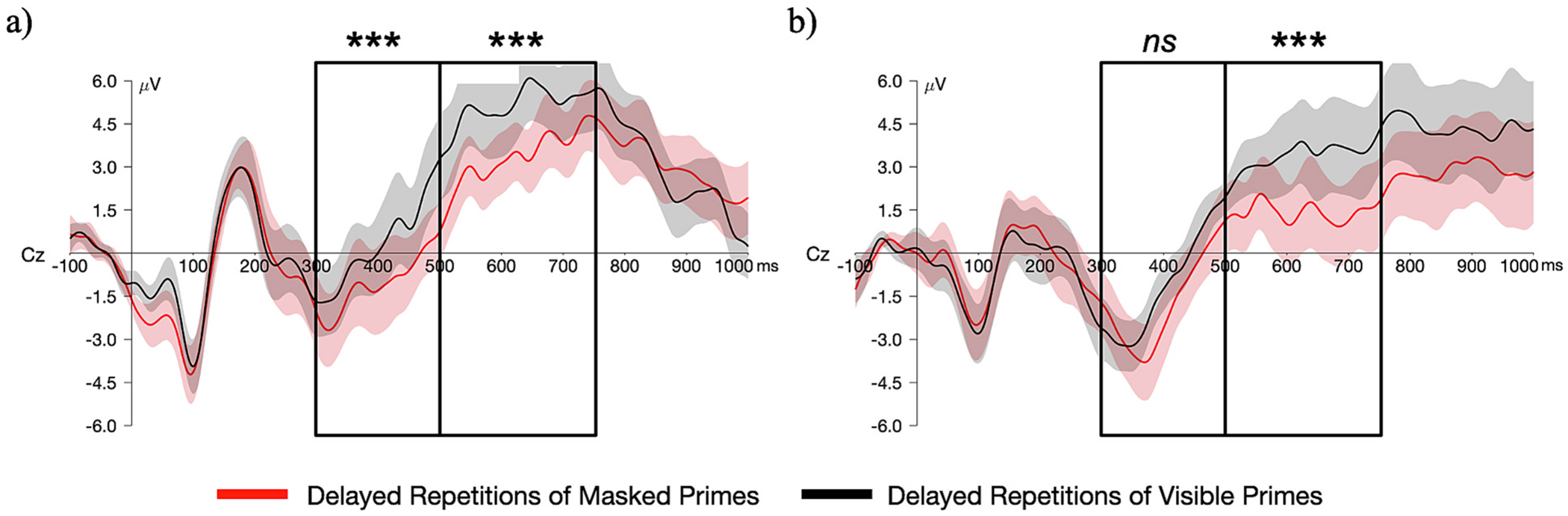
Grand average ERP waveforms at electrode Cz for a) Controls and b) PWA elicited by delayed repetitions of masked primes (red) and delayed repetitions of visible primes (black). Shaded regions indicate ± 1 standard error of the mean. Vertical boxes highlight the analyzed N400 and LPC time windows. Statistical significance is denoted by asterisks (*** *p* < 0.001; *ns* = not significant).

To directly examine how responses to the primes varied depending on whether the priming effects stemmed from implicit processes alone or from a combination of implicit and explicit processes, we compared mean amplitudes for Delayed Repetitions of Masked Primes and Delayed Repetitions of Visible Primes (see Fig. 9). Controls showed significantly more negative mean amplitudes for Delayed Repetitions of Masked Primes than for Delayed Repetitions of Visible Primes (*β* = −4.88, *SE* = 1.02, *t*(2822) = −4.79, *p* < 0.001), while the difference between these target types was not significantly different for PWA (*β* = −1.34, *SE* = 0.98, *t*(2822) = −1.36, *p* = 0.173). This divergent pattern was supported by a significant interaction of Delayed Repetitions of Visible Primes versus Delayed Repetitions of Masked Primes x Group (*β* = 7.08, *SE* = 2.83, *t*(2822) = 2.50, *p* = 0.012). No other interactions were significant (*p*s > 0.05).

**Fig. 10.**
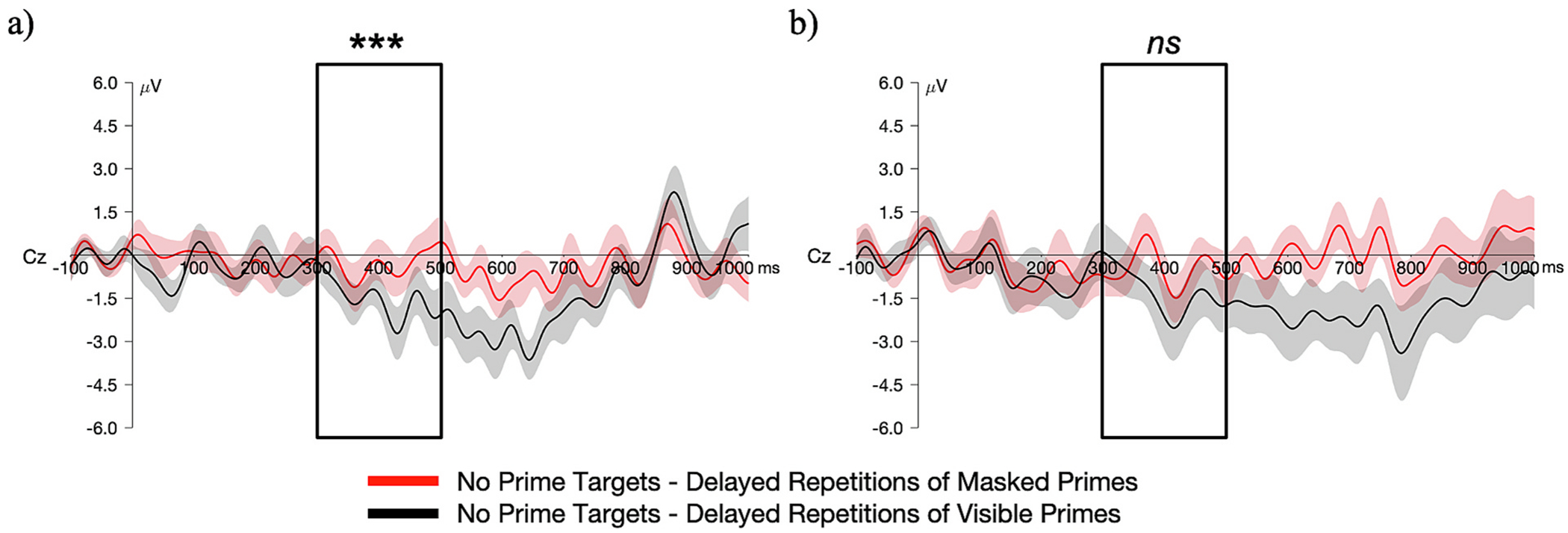
Grand average difference waves at electrode Cz for a) Controls and b) PWA, comparing delayed repetitions of masked primes (red) and delayed repetitions of visible primes (black). More negative deflections indicate larger priming effects. Shaded regions indicate ± 1 standard error of the mean. Vertical boxes highlight the analyzed N400 time window. Statistical significance is denoted by asterisks (*** *p* < 0.001; *ns* = not significant).

To confirm these amplitude differences reflected differences in priming effects, we again examined the magnitude of the priming effects using mean amplitudes of difference waves. Consistent with the initial findings, Controls showed greater priming effects for Delayed Repetitions of Visible Primes than for Delayed Repetitions of Masked Primes (*β* = −4.88, *SE* = 0.90, *t*(2257) = −5.45, *p* < 0.001), while PWA did not show a significant difference (*β* = −1.34, *SE* = 0.86, *t*(2257) = −1.55, *p* = 0.121). The interaction of Delayed Repetitions of Visible Primes versus Delayed Repetitions of Masked Primes x Group remained significant (*β* = 7.08, *SE* = 2.49, *t*(2257) = 2.85, *p* = 0.004; see Fig. 10). There were no other significant interactions (*p*s > 0.05).

## 4. Discussion

In this study, we examined the time course of automatic spreading activation in PWA and age-matched adults with typical language, using EEG during a priming task with both masked and visible primes. Results indicated that PWA exhibited extended maintenance of activation rather than the predicted delay in its initial spread. In addition, there was unexpected evidence that suggests that PWA might also be less able to engage explicit processing to the same extent as their typical peers. Together, these findings imply that in PWA, activation of a word may be affected not only by prolonged activation maintenance, but also by impaired integration of activation from bottom-up implicit processes and top-down explicit processes, potentially leading to a reliance on the impaired implicit process alone.

### 4.1. Experimental design validation

Converging evidence from the probe identification data, behavioral recognition task, P300 analyses, and some of the LPC analyses (see Supplemental Materials for detailed data and further discussion) suggests that both groups of participants understood and were engaged with the task throughout its completion. Importantly, these data also confirm that participants did not consciously perceive, encode, or process the masked primes, consistent with Misra and Holcomb’s (2003) data from young adults with typical language. This supports our conclusion that the priming effects observed were the product of implicit, rather than explicit, processing.

### 4.2. Automatic spreading activation in PWA

We used behavioral *and* ERP priming effects as indices of automatic spreading activation. Interestingly, neither the Controls nor the PWA showed behavioral priming effects via their reaction times. Although less common in unimpaired adults, it is not unprecedented for PWA to fail to show behavioral priming effects from masked primes, particularly when the ISI is near 500 ms (Silkes & Rogers, 2012), as in the present study. Moreover, implicit effects often occur even when they are not reflected in the explicit behavioral responses they underlie (e.g., Abel et al., 2020; Pankonin et al., in press). Despite the lack of behavioral priming effects, we found robust N400 priming effects. While these divergent findings might seem at odds, they exemplify the value of investigating the cognitive processes underlying behavioral responses isolated from conscious influences.

To directly address our research question regarding the time course of spreading activation, we conducted a series of analyses examining the N400 priming effects for immediate repetitions of masked primes and delayed repetitions of masked and visible primes. We predicted that PWA would exhibit delayed spreading activation, showing priming effects for delayed repetitions of masked primes but not for immediate ones, while Controls would show the opposite pattern. Contrary to this prediction, both Controls and PWA showed priming effects for immediate repetitions of masked primes. In fact, the magnitude of this effect exceeded that of the delayed repetitions of masked primes. This finding suggests that PWA in our sample had no significant delay in initiating the spread of activation, or that any such delay was not enough to noticeably affect the activation level reached by the time the target appeared.

However, group differences emerged with the delayed repetitions of masked primes: PWA continued to show priming effects whereas Controls did not. These effects in PWA were weaker than those elicited by immediate repetitions of masked primes, indicating that the activation from the primes for these targets had decreased but not yet returned to baseline. Instead, the activation remained elevated enough to facilitate processing of the target for an unusually extended period (beyond 25 s for some participants). As such, our findings suggest that, although our sample of PWA did not initially spread activation more slowly, they nonetheless showed an alteration in their time course of spreading activation; specifically, they showed a deficit in passive decay rates or the inhibition of activation.

#### 4.2.1. Inhibitory control as an explanatory mechanism

Much of the research on activation patterns in aphasia has found evidence for slowed automatic spreading activation (e.g., Ferrill et al., 2012; Prather et al., 1992; Silkes et al., 2020; Silkes & Rogers, 2012) and/or accelerated activation decay (e.g., Martin et al., 2018, 2022; Martin & Dell, 2019; Prather, 1994; Prather et al., 1997; Silkes & Rogers, 2012). However, we are not the first to have found evidence for abnormally prolonged activation (e.g., Prather et al., 1997; Prather, 1994). Notably, Prather and colleagues found that some PWA, particularly those with fluent aphasia, like the majority of our sample of PWA, initiate activation at a typical rate but show a slowed decline in activation. They proposed that this difference is due to deficits in inhibitory control.

Inhibiting activation is as critical to lexical retrieval as initiating and transmitting it; without sufficient inhibitory forces to counteract excitatory ones, too many representations may remain simultaneously active (e.g., Berg & Schade, 1992; Blomquist & McMurray, 2023; Dell, 1986; Dell & O’Seaghdha, 1992). Consequently, numerous active lexical items compete for selection, hindering retrieval (e.g., Berg & Schade, 1992; Blomquist & McMurray, 2023; Dell, 1986; Dell & O’Seaghdha, 1992; Hasher et al., 1991). This competition could manifest as word retrieval difficulties, as more cognitive resources and/or time may be needed to resolve competition and retrieve the intended lexical item (e.g., Botezatu & Mirman, 2019; Dell et al., 1997; Foygel & Dell, 2000; Hasher et al., 1991; Pompon et al., 2015; Schwartz et al., 1994).

Our data are consistent with the idea of increased lexical competition resulting from prolonged activation, as evidenced by the pattern of priming effects for the immediate repetitions of masked primes across two different baseline conditions. Specifically, the priming effect was smaller when the unprimed baseline target had an unrelated word in the prime position than when it had no word there. This pattern suggests that the unrelated prime word remained active and interfered with processing the target when it was encountered (Bjork, 1989; Conway & Engle, 1994). Some interference is expected, especially in older individuals (Hasher et al., 1991; Hasher & Zacks, 1988; Kane et al., 1994), and Controls did show reduced priming following an unrelated prime compared to no prime; however, the reduction was less pronounced than in PWA, suggesting that age-related interference alone does not fully explain this pattern for PWA. Furthermore, prior work has documented inhibitory deficits in PWA for a variety of cognitive and linguistic processes (e.g., Frauenfelder et al., 2001; Hamilton & Martin, 2005; Lim et al., 2012; Pompon et al., 2015; Wiener et al., 2004), supporting the hypothesis that an impairment in inhibitory control could contribute to anomia in PWA.

#### 4.2.2. Patterns of activation maintenance and decay

Importantly, our findings of abnormally prolonged activation are not necessarily contradictory to prior studies’ findings of delayed spread of activation or its accelerated decay. Both activation transmission and representation integrity play a role in the time course of automatic spreading activation, and multiple forms of processing impairment may co-occur (Martin et al., 2018, 2022; Martin & Dell, 2019; Prather, 1994; Prather et al., 1997). Our experimental design may have been less sensitive to detecting very brief delays in spreading activation and/or our sample of PWA may have predominantly consisted of individuals with prolonged activation maintenance rather than delayed spread and/or accelerated decay of activation. Further work is needed to explore variability across PWA, including the underlying mechanisms supporting these processing differences (e.g., active inhibition versus passive decay of activation).

#### 4.2.3. Evidence for impaired integration of explicit information

Another interesting finding is that, unlike the control group, the PWA showed equally strong priming effects for delayed repetitions of visible primes and delayed repetitions of masked primes. This pattern was unexpected, as masked primes generally yield weaker effects than visible primes (e.g., Forster & Davis, 1984; Kiefer, 2002; Marcel, 1983; Misra & Holcomb, 2003) since a visible prime allows top-down processing that may reinforce the bottom-up, implicit activation of the target. Consistent with this typical pattern, the control adults showed much larger priming effects for visible than masked primes. In contrast, PWA exhibited no such difference: their neural response to a consciously perceived prime was no stronger than their response to a masked prime that never reached conscious awareness.

This finding suggests that the PWA may have been less able to capitalize on the stronger activation that accompanies an explicit, visible prime. This impairment may reflect a reduced ability to rapidly integrate explicit information and reinforce the initial bottom-up activation (e.g., Dell, 1986; Foygel & Dell, 2000; Nadeau, 2001). Without this reinforcement, PWA would be dependent on automatic, implicit processing, leading the visible primes to produce priming effects comparable in magnitude to masked primes. This interpretation aligns with prior aphasia research repeatedly showing that PWA can often access and use information implicitly that they cannot demonstrate explicitly (e.g., Coslett & Saffran, 1994; Mimura et al., 1996; Revonsuo, 1995; Roberts et al., 2010).

### 4.3. Implications

The findings of this study suggest that prolonged maintenance of activation and deficits in integrating activation from implicit and explicit processes are likely mechanisms underlying language processing impairments in aphasia, particularly anomia. These multiple impairments may help explain why anomia is a common symptom in PWA across varied profiles and have implications for both our understanding and clinical treatment of the language processing impairments PWA experience, specifically regarding word retrieval. The finding of reduced integration is particularly novel and warrants further investigation to clarify the specific mechanisms involved.

These results also suggest that repeated exposure to a word can strengthen activation in PWA, albeit for a longer time due to longer-lasting activation than in a typical system. This knowledge can inform aphasia treatments by guiding the way in which stimuli are presented and reinforced in treatment contexts, such as by manipulating the timing or the tasks involved, so that PWA can maximize engagement of their lexical networks. For example, treatment might involve more time between trials to allow activation to adequately dissipate or, as in repetition priming treatment paradigms (e.g., Silkes, 2023; Tabrizi, Walton et al., 2022), may leverage prolonged activation to target lexical connections. In addition, identifying these impaired mechanisms creates opportunities for developing treatments that directly address them, such as interventions that aim to address the timing of automatic spreading activation through manipulating stimulus–response intervals (e.g., Conroy et al., 2018; Kalinyak-Fliszar et al., 2011; Martin et al., 2021).

### 4.4. Study limitations

Most of the limitations of this study stem from deliberate experimental design choices made to ensure experimental control while minimizing introducing additional complexity. These decisions now offer directions for future research.

First, we were unable to examine electrophysiological responses to targets that were immediate repetitions of visible primes, as the stimulus lists were not constructed to include any trials in which a visible prime immediately preceded a target (i.e., within a single trial). Consequently, all visible prime-target relationships involved delays. Given the complexity of the stimulus lists and overall study design, it was not feasible to add another condition with immediate repetitions of visible primes. However, incorporating such a condition in future studies could provide valuable insight into how visible primes are processed in both populations.

We also could not separate the masked and visible delayed prime conditions by the different numbers of intervening trials due to how the EEG data were organized during acquisition. This limited our ability to analyze the effects of interest at this level of granularity. Additionally, while we held the SOA at 500 ms to maintain control with the several other timing manipulations, delays in spreading activation may have occurred and been resolved within this interval. To better understand the precise onset and maintenance duration of spreading activation in PWA, future studies should examine priming effects at systematically varied SOAs.

Another set of limitations concerns the ERP component analyses. Given the natural individual variability in lesion location and size among PWA, we did not control for these factors or their potential relation to electrode site selection. However, as signal localization was beyond the study’s scope, and since the effects of interest are usually distributed rather than focal, examining broader cortical fields was a reasonable approach that still yielded meaningful and interpretable results. Future studies incorporating lesion characteristics could offer further insights into the influence of lesion variability.

We also did not separate our analyses of PWA by fluency level, aphasia or anomia severity, or other classifications (e.g., “classical” aphasia subtypes). All PWA experience anomia to some degree, regardless of their overall language profile, and the aim of this study was to understand the broader mechanisms underlying this particular ubiquitous symptom across aphasia profiles and severities rather than explore subgroup differences. The classical aphasia classifications, in particular, are imprecise and, therefore, of limited utility (e.g. Tremblay and Dick, 2016), and our sample size lacked sufficient statistical power for subgroup comparisons. Nonetheless, future studies could investigate whether the timing of spreading activation in PWA varies by subgroup or individual characteristics.

Finally, we took a conservative approach to participant inclusion, excluding all data from participants with substantial EEG artifacts rather than risk introducing noise by including the limited, seemingly “un-contaminated” data from these participants. While this notably reduced the final sample size, it ensured the reliability of the ERP responses. In addition, we not only identically replicated a published procedure (Misra & Holcomb, 2003), but we also incorporated extensive experimental validation measures and used an EEG recording system that met all standards to ensure data quality. Thus, despite the small sample size, our findings remain reliable and meaningfully contribute to understanding the time course of spreading activation in PWA.

### 4.5. Conclusion

This study’s findings add to the evidence that aphasia involves altered temporal dynamics of automatic spreading activation in the language system. Specifically, these data suggest that abnormally prolonged activation is a mechanism that may underlie language processing impairments in PWA, including word retrieval. These data also indicate that an inability to adequately integrate the activation from explicit and implicit processes might also contribute to aphasia, as PWA failed to benefit from the additional activation supplied by explicitly processing stimuli that reached conscious awareness, and instead likely relied on their atypical implicit processes. Together, these insights offer a more nuanced understanding of aphasia and inform the development of more effective aphasia treatment methods and directions for future research.

## Supplementary Material

1

## Figures and Tables

**Table 1:** Planned comparisons and the ERP components analyzed, purpose, predictions for each comparison based on prior literature on typical participants, and interpretation. Comparisons 1–2 and the LPC results for comparisons 3–6 are validation measures, not germane to the research questions, so those data are reported in detail in the Supplemental Materials.

#	Comparison	ERP component(s) analyzed	Purpose of comparison	Prediction(s) based on typical literature	Interpretation(s)
1	Masked prime probes vs. masked critical primes	P300	Measure of masking effectiveness	No difference between stimulus types expected.	Primes were effectively masked.
2	Unprimed visible probe targets vs. unprimed visible critical targets	P300	Measure of task comprehension and engagement	Enhancement expected for unprimed visible probe targets.	Participant could differentiate animal probe from critical non-animal words and were attending to the stimuli.
3	Delayed repetitions of visible primes vs. unprimed targets	N400	Validation of experimental protocol effectiveness	Attenuation (i.e., priming) expected for delayed repetitions of visible primes.	The experimental protocol was adequate to elicit the expected effects, in both groups, from the strongest form of priming.
		LPC	Measure of conscious perception of visible non-animal words	Enhancement expected for delayed repetitions of visible primes.	Participant has perceived the prime and recognized its repetition.
4	Immediate repetitions of masked primes vs. unprimed targets	N400^a^	Examine speed of automatic spreading activation via presence / absence of automatic semantic processing	Attenuation (i.e., priming)expected for immediate repetitions of masked primes.	Typical initial spread of activation. If PWA do not meet this prediction (i.e., do not show priming), this would indicate delayed automatic spreading activation.
		LPC	Measure of masking effectiveness	No difference between stimulus types.	Primes were effectively masked.
5	Delayed repetitions of masked primes vs. unprimed targets	N400 ^a^	Examine speed of automatic spreading activation via presence/absence of automatic semantic processing	Weak (if any) attenuation (i.e., weak, or no, priming) expected for delayed repetitions of masked critical primes.	Activation has appropriately decayed over time. If PWA do not meet this prediction (i.e., show priming effects), that would indicate delayed automatic spreading activation or, if priming has occurred for immediate repetitions (Comparison #4), prolonged maintenance of activation.
		LPC	Measure of masking effectiveness	No difference between stimulus types expected.	Primes were effectively masked.
6	Delayed repetitions of masked primes vs. delayed repetitions of visible primes	N400 ^a^	Examine magnitude of priming effects.	Greater attenuation (i.e., priming) expected for delayed repetitions of visible primes than delayed repetitions of masked primes.	Effective integration of explicitly and implicitly processed information allowed for stronger priming than solely implicitly processed information.
		LPC	Measure of masking effectiveness	Enhancement expected for delayed repetitions of visible primes.	Primes were effectively masked, and visible targets were consciously perceived.

a Because predictions presented here are based on the typical literature, these critical comparisons are the most likely to not have predictions met by PWA due to differences in automatic spreading activation.

**Table 2:** Individual participant characteristics for PWA.

Participant ID	Sex	Age	Etiology	Years Since Stroke	Fluency Classification	WAB AQ	BNT	RCPM
A301	F	33	Aneurysm/L CVA	2.85	Fluent	83.5	41	35
A302	M	68	L CVA	9.82	Non-fluent	80.5	47	33
A304	F	68	L CVA	7.86	Fluent	89.8	42	35
A305	M	54	L CVA	7.86	Non-fluent	70.8	21	23
A306	M	51	L CVA	2.93	Fluent	87.5	37	33
A307	M	70	L CVA	1.10	Fluent	94.7	35	23
A312	F	68	L CVA	10.09	Fluent (but apraxic)	90	48	35
A314	M	63	4.5″ L CVA	2.03	Fluent	48.8	2	30
A317	M	61	L CVA	7.32	Fluent	47.9	0	34
A318	M	62	Large L CVA	5.52	Non-Fluent (and apraxic)	22.8 (verbal/ written)	37 (written)	34
A319	M	72	L CVA	12.03	Non-fluent	62.5	14	30
A320	M	74	L CVA	7.55	Fluent	40.3	2	33
A322	F	51	L CVA	1.75	Fluent	74.3	41	29
A323	M	50	L CVA	0.55	Non-fluent	58.7	8 (verbal) 24 (written)	32

*Note.* All etiologies are based on participant report. Written responses were permitted for some participants due to severe apraxia of speech. Parenthetical notes specify whether the score reflects entirely verbal responses, entirely written responses, or a mix of both response modalities. Fluency classification is based on WAB criteria. PWA = people with aphasia; M = male; F = female; L CVA = left cerebrovascular accident; WAB AQ = Western Aphasia Battery Aphasia Quotient; BNT = Boston Naming Test; RCPM = Raven’s Coloured Progressive Matrices.

**Table 3:** A representative sample of a stimulus list and designation of the various stimulus conditions.

prime	TARGET	Prime Condition	Target Condition
	POUCH	blank screen	no-prime target
harp	TWIN	masked prime	unrelated-prime target
	MOOSE	blank screen	unprimed animal probe
posy	POSY	masked prime	immediate repetition of masked prime
	HARP	blank screen	delayed repetition of masked prime ^a^
	POUCH	blank screen	delayed repetition of visible prime ^a^
shark	GONG	masked prime probe	filler target
	SHARK	blank screen	primed animal probe ^a^

*Note.* Each row represents one trial, and the trials are arranged in the order they would be presented to create the stated conditions.

a Primed animal probes and both types of delayed repetitions were pseudorandomly varied to occur from one to eight trials following their prime, with an average of four intervening trials.

**Table 4:** Response accuracy for probe identification.

	Controls M	SD	PWA M	SD
Prime probes	0.11	0.12	0.09	0.15
Primed probe targets	0.73	0.28	0.75	0.24
Unprimed probe targets	0.80	0.19	0.72	0.21

## Data Availability

Materials, data, and analysis code for this study are available at https://osf.io/2rqny/

## Appendix A. Supplementary data

Supplementary data to this article can be found online at https://doi.org/10.1016/j.clinph.2025.2110972.

## Abbreviations:

PWA: People with aphasia
