# Supplementary material for: Timing of implicit processes in aphasia: An event-related potential investigation of masked priming effects
Source: Clin Neurophysiol. Author manuscript; Available in PMC 2026 Jul 10. (PMC13352526; doi:10.1016/j.clinph.2025.2110972)
Supplement: 1 [file NIHMS2181300-supplement-1.docx]

**Supplemental Materials**

### S1. Experimental Design Validation Measures

The purpose of the various experimental design validation measures was to verify that the masked primes were effectively masked and, thus, were not consciously perceived, encoded, and/or otherwise processed in any way. This was necessary so that we could confidently attribute any priming effects from the masked primes to solely automatic, implicit processes. We also wanted to confirm that we had achieved our goal with the words in the experimental priming task that we intended to be consciously perceived, encoded, and/or otherwise processed (i.e., the visible words). As such, in addition to the behavioral priming measures and probe detection task described and reported in the main text, we examined behavioral responses on a recognition task and neural responses indexing conscious processing to the various stimulus types of the experimental priming task. Only information unique to the measures not included in the main text is detailed here.

#### S1.1. Stimuli and Procedure

**S1.1.1. Recognition Task**

Immediately after completing the experimental priming task, each participant was given a recognition test to assess their conscious awareness of the content of the masked critical words that had been presented during the experimental trials. For this task, they were presented with a list of 150 printed non-animal words in one of eight random orders. Fifty of those words were the critical words that were never presented in the experimental priming task, which served as foils. Half of the remaining 100 were masked primes of the unrelated-prime targets (*n* = 50), and half were no-prime targets (i.e., visible critical targets that were presented without a prime) that only appeared once in the entire experimental priming task (*n* = 50). Participants were asked to circle any of the words that they had seen on the computer screen during the experiment.

**S1.2. Data Analysis**

We used the same analytical approach for the behavioral and ERP data described in Section 2.6 to analyze the behavioral responses from the recognition task and the mean amplitudes from the P300 and LPC time windows. We analyzed response accuracy in the recognition task just as we analyzed response accuracy in the experimental priming task, except that the Stimulus Type factor’s levels were Masked Words, Visible Words, and Foils for the recognition task data analysis.

 Time window and electrode selections for the P300 and LPC were based on previous research studies examining these ERP components (e.g., Geal-Dor et al., 2006; Haebig et al., 2018; Hill et al., 2002; Misra & Holcomb, 2003; van Petten et al., 1991). For the P300 analyses, we obtained mean amplitudes from 400 to 1000 ms post-stimulus onset from the same 21 electrodes specified in Section 2.6 for masked animal prime probes, masked primes, probe targets, and critical targets. We analyzed the data for the primes and targets separately, using the same linear mixed-effects (LME) model as used for analyzing the N400 mean amplitudes but with a two-level factor of Stimulus Type (Prime Probe, Critical Prime; Probe Target, Critical Target) instead of a five-level factor of Target Type.

For the LPC, we obtained mean amplitudes from 500 to 750 ms post-target onset from a more restricted sample of electrodes, specifically C3, Cz, C4, CP3, CPz, CP4, P3, Pz, P4, PO3, POz, PO4, O1, Oz, and O2. Mean amplitudes from the LPC window were analyzed using the same LME model for the N400 mean amplitude analysis. Examining the magnitude of priming effects for the P300 or LPC ERP components was beyond the scope of this study.

### S2. Results and Discussion

#### S2.1. Recognition Task: Verifying Masking Effectiveness via Behavior

Both controls and PWA demonstrated the same pattern of recognition: all participants recognized a significantly greater proportion of visible words than masked words (Controls: *β* = 0.37, *SE* = 0.06, *t*(73) = 5.78, *p* < .001; PWA:  *β* = 0.18, *SE* = 0.06, *t*(73) = 2.94, *p* = .004) and at least trended towards recognizing a greater proportion of visible words than foils (Controls: *β* = 0.93, *SE* = 0.13, *t*(73) = 5.78, *p* < .001; PWA:  *β* = 0.31, *SE* = 0.12, *t*(73) = 2.53, *p* = .014), while recognition of the masked words and foils did not significantly differ (Controls: *β* = 0.18, *SE* = 0.32, *t*(73) = 1.41, *p* = .164; PWA: *β* = -0.05, *SE* = 0.12, *t*(73) = -0.41, *p* = .681; see Table S1). The Controls also showed a greater difference in accuracy between recognizing visible words and foils than the PWA did, as reflected by the significant interaction of Visible Words versus Foils x Group (*β* = 1.23, *SE* = 0.36, *t*(73) = 3.42, *p* = .001). Thus, these findings indicate that, for both groups, the masked primes were not encoded while the visible stimuli were, supporting our assumption that the masked primes were, indeed, effectively masked while the visible words were consciously perceived and processed.

Supplemental Table 1. Proportion of responses in the recognition task.

|  | Controls | | PWA | |
| --- | --- | --- | --- | --- |
|  | *M* | *SD* | *M* | *SD* |
| Visible Words | 0.28 | 0.2 | 0.25 | 0.27 |
| Masked Words | 0.12 | 0.14 | 0.16 | 0.16 |
| Foils | 0.08 | 0.11 | 0.16 | 0.18 |

#### S2.2. P300: Verifying Masking Effectiveness via ERPs

The P300 is larger (i.e., positively enhanced) when a participant *consciously* detects a task-relevant event (Luck, 2012; Polich, 2007), such as the animal-name probes in our experimental priming task. Because non-animal words are not relevant to the task of responding whenever an animal name is detected, we did not expect any P300 enhancement to the critical non-animal words. Furthermore, because the P300 only occurs when task-relevant stimuli reach conscious awareness, the masked animal prime probes were not expected to elicit a P300 enhancement even though they were relevant to the semantic categorization task; if the masking prevented them from being consciously received, they could not be recognized as relevant. Thus, we only expected increased amplitudes in the P300 window for probes that reached conscious awareness, which should only be the visible probe words (i.e., animal targets).

We first compared the mean amplitudes for masked prime probes and masked primes. We found that there were no significant differences between the mean amplitudes for these two stimulus types for either group when corrected for multiple comparisons (Controls: *β* = 0.38, *SE* = 0.19, *t*(1127) = 2.07, *p* = .038; PWA: *β* = -0.05, *SE* = 0.18, *t*(1127) = -0.30, *p* = .761), providing evidence that all masked primes were effectively masked and not consciously perceived or processed. We also compared the mean amplitudes during the P300 window for probe targets and critical targets. Both groups demonstrated a significantly more positive mean amplitudes for probe targets compared to critical targets (Controls: *β* = -0.87, *SE* = 0.20, *t*(1127) = -4.42, *p* < .001; PWA: *β* = -0.95, *SE* = 0.19, *t*(1127) = -4.98, *p* < .001), indicating the visible animal probe targets reached participants’ conscious awareness and were recognized as relevant. There were no significant interactions of any Stimulus Type with Group (*p*s > .05). Thus, our results verify that targets and primes were perceived or not, respectively, as intended, supporting our interpretations of the priming effects as reflecting implicit, automatic processing. See Supplemental Figures 1 and 2 for the ERP waveforms included in this analysis.

**Supplemental Figure 1*.*** Grand average ERP waveforms at electrode Pz for a) Controls and b)

PWA elicited by prime probes (red) and masked primes (black). Shaded regions indicate ± 1

standard error of the mean. Vertical boxes highlight the analyzed P300 time window. Statistical

significance is denoted by asterisks (*ns* = not significant).


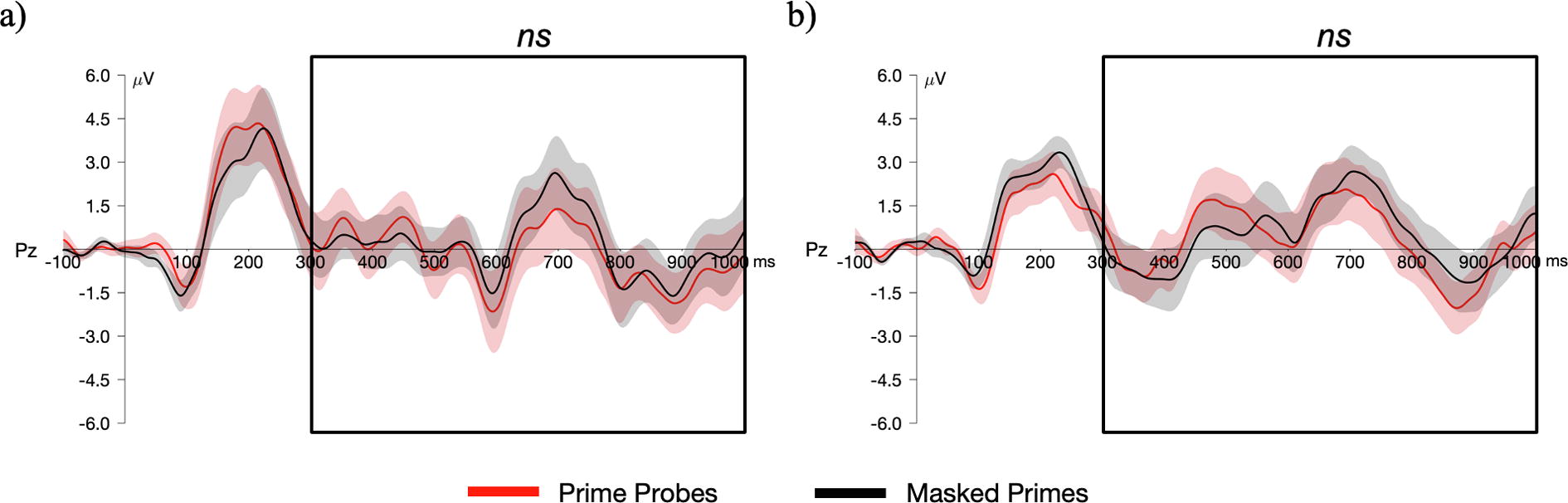


**Supplemental Figure 2.** Grand average ERP waveforms at electrode Pz for a) Controls and b)

PWA elicited by unprimed probe targets (red) and unprimed critical targets (black). Shaded

regions indicate ± 1 standard error of the mean. Vertical boxes highlight the analyzed P300 time

window. Statistical significance is denoted by asterisks (*** *p* < .001).


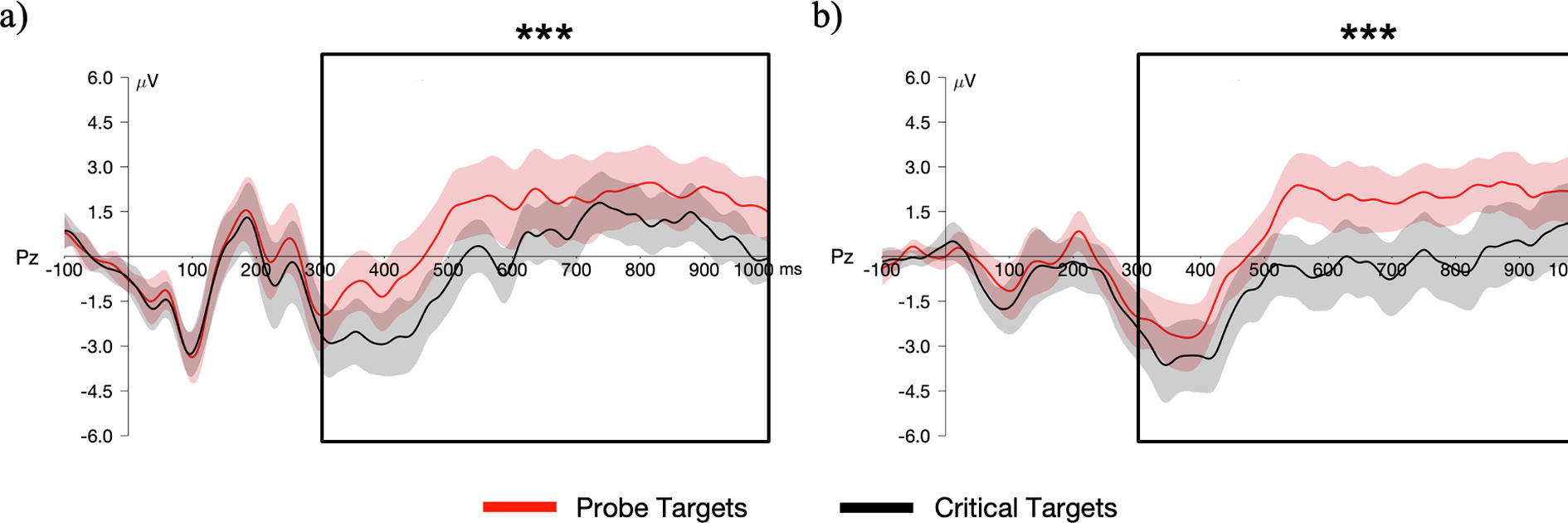


**S2.3. LPC: Verifying Priming Effects Reflect Implicit or Explicit Processing**

The LPC is sensitive to repetition (e.g., Rugg, 1990) in that it is positively enhanced when a stimulus is repeated compared to the first time the stimulus is presented. Critically, this enhancement is thought to only occur when the stimulus is consciously perceived at both presentations, as it relies on the conscious retrieval of episodic memories (van Petten et al., 1991). We expected both controls and PWA to demonstrate the same LPC effects because the LPC reflects explicit processes that are outside the lexical-semantic system. The LPC also assists in the interpretation of any N400 effects that are observed. If an N400 priming effect is observed in the absence of an enhanced LPC, it is safe to assume that the N400 effect was the result of implicit processing rather than conscious processing of the prime. Based on Misra and Holcomb’s (2003) findings, we expected both groups to show only increased mean amplitudes during the LPC window for delayed repetitions of visible primes.

**S2.3.1. Delayed Repetitions of Visible Primes versus Unprimed Targets**

Analyses of mean amplitudes during the LPC time window revealed that, as expected, both the Controls (*β* = 11.97, *SE* = 1.12, *t*(2012) = 10.71, *p* < .001) and PWA (*β* = 7.07, *SE* = 1.08, *t*(2012) = 6.56, *p* < .001) showed more positive mean amplitudes for Delayed Repetitions of Visible Primes than No-Prime Targets (see Figure 4 in the main text).

**S2.3.2. Delayed Repetitions of Visible Primes versus Delayed Repetitions of Masked Primes**

As expected, Delayed Repetitions of Visible Primes also had more positive-going mean amplitudes than Delayed Repetitions of Masked Primes for the Controls (*β* = -8.59, *SE* = 1.12, *t*(2012) = -7.68, *p* < .001) and for the PWA (*β* = -5.97, *SE* = 1.08, *t*(2012) = -5.55, *p* < .001; see Figure 7 in the main text).

**S2.3.3. Immediate Repetitions of Masked Primes versus Unprimed Targets**

Following our predictions, there was no difference between mean amplitudes for Immediate Repetitions of Masked Primes and Unrelated-Prime Targets for Controls (*β* = -1.43, *SE* = 1.12, *t*(2012) = -1.28, *p* = .201). However, PWA showed a different, unexpected pattern of more positive deflections during the LPC window for Immediate Repetitions of Masked Primes (*β* = -4.19, *SE* = 1.08, *t*(2012) = -3.89, *p* < .001). Additionally, contrary to our expectations, both the Controls (*β* = 4.40, *SE* = 1.12, *t*(2012) = 3.93, *p* < .001) and PWA (*β* = 5.69, *SE* = 1.08, *t*(2012) = 5.28, *p* < .001) showed more positive mean amplitudes for Immediate Repetitions of Masked Primes when No-Prime Targets were used as the unprimed target baseline. See Figure 3 in the main text for the ERP waveforms of these comparisons.

**S2.3.4. Delayed Repetitions of Masked Primes versus Unprimed Targets**

PWA showed the expected pattern, failing to show any differences between Delayed Repetitions of Masked Primes and No-Prime Targets (*β* = 1.09, *SE* = 1.08, *t*(2012) = 1.01, *p* = .310). Controls, however, showed more positive mean amplitudes for Delayed Repetitions of Masked Primes (*β* = 3.38, *SE* = 1.12, *t*(2012) = 3.03, *p* = .002; see Figure 4 in the main text). Controls and PWA’s different LPC responses to Delayed Repetitions of Masked Primes was also indicated by a significant interaction of Delayed Repetitions of Masked Primes versus No-Prime Targets x Group (*β* = -9.81, *SE* = 3.10, *t*(2012) = -3.16, *p* = .002).

**S2.3.5. Immediate Repetitions of Masked Primes versus Delayed Repetitions of Masked Primes**

In line with our predictions, there was no difference between mean amplitudes of these two stimulus types for Controls (*β* = 1.01, *SE* = 1.12, *t*(2012) = 0.90, *p* = .366; see Figure 5 in the main text). Unlike the Controls, though, the PWA unexpectedly showed more positive deflections during the LPC window for Immediate Repetitions of Masked Primes versus Delayed Repetitions of Masked Primes (*β* = 4.60, *SE* = 1.08, *t*(2012) = 4.27, *p* < .001; see Figure 5 in the main text). The significant interaction of Immediate Repetitions of Masked Primes versus Delayed Repetitions of Masked Primes x Group (*β* = 7.17, *SE* = 3.10, *t*(2012) = 2.31, *p* < .001) also reflected the groups’ contrasting response patterns.

**S2.3.6. Discussion of Visible Prime Comparisons: Evidence for Explicit Processing**

As expected, both groups showed more positive deflections during the LPC time window for delayed repetitions of visible primes than for no-prime targets or delayed repetitions of masked primes. This finding is consistent with Misra and Holcomb’s (2003) results and suggests that visible primes were consciously perceived both when they were first presented as a prime and when they were presented again as a target. It also shows that delayed repetitions of visible primes elicited a stronger effect than delayed repetitions of masked primes, as would be expected if the masked primes were never consciously perceived at their first presentation as primes, thus preventing any conscious recollection when they were encountered at their second presentation as targets.

**S2.3.7. Discussion of Masked Prime Comparisons: Mixed Evidence for Implicit Processing**

 The LPC analyses of the immediate and delayed repetitions of masked primes resulted in a mix of expected and unexpected findings. We had only predicted increased positivity to primes when they were consciously perceived at their first (and subsequent) encounter. All the masked primes were allegedly imperceptible, which should have prevented any LPC effect from occurring. However, in several cases for both the Controls and PWA, we found an LPC effect for masked primes.

While these findings imply that participants consciously detected and processed the masked primes, the other data that show a clear distinction between detection of masked versus visible words (i.e., the probe detection task results, the recognition task results, and the P300 results) suggest otherwise. Thus, the effect captured during the LPC time window might reflect a process other than explicit recognition of a previously consciously perceived stimulus. For example, given that the LPC time window began at the same time point the N400 window ended and is subsumed within the P300 time window, it could be that we are capturing an extension of the N400 effect and/or an inextricable combination of components. Indeed, previous research has found that the N400 effect is often prolonged in both PWA and unimpaired older adults (e.g., Chang et al., 2016; Kutas & Iragui, 1998; Stalpaert et al., 2021), which could have also occurred in our sample. This possibility would require a detailed analysis beyond the scope of this study but is worthy of future research.

In sum, our unexpected findings are tempered by both the behavioral and other ERP data presented as part of this study and the extant literature on the LPC, reducing concerns that these LPC responses indicate the priming effects were due to something more than implicit mechanisms. Ultimately, the data presented here largely confirm the supposition that the masked priming effects observed in this study were the product of implicit, rather than explicit, processing mechanisms.

**Supplemental References**

Chang, C.-T., Lee, C.-Y., Chou, C.-J., Fuh, J.-L., & Wu, H.-C. (2016). Predictability effect on N400 reflects the severity of reading comprehension deficits in aphasia. *Neuropsychologia*, *81*, 117–128. https://doi.org/10.1016/j.neuropsychologia.2015.12.002

Geal-Dor, M., Goldstein, A., Kamenir, Y., & Babkoff, H. (2006). The effect of aging on event-related potentials and behavioral responses: Comparison of tonal, phonologic and semantic targets. *Clinical Neurophysiology*, *117*(9), 1974–1989. https://doi.org/10.1016/j.clinph.2006.05.024

Haebig, E., Leonard, L., Usler, E., Deevy, P., & Weber, C. (2018). An initial investigation of the neural correlates of word processing in preschoolers with specific language impairment. *Journal of Speech, Language, and Hearing Research*, *61*(3), 729–739. https://doi.org/10.1044/2017_JSLHR-L-17-0249

Hill, H., Strube, M., Roesch-Ely, D., & Weisbrod, M. (2002). Automatic vs. Controlled processes in semantic priming—Differentiation by event-related potentials. *International Journal of Psychophysiology*, *44*(3), 197–218. https://doi.org/10.1016/S0167-8760(01)00202-1

Kutas, M., & Iragui, V. (1998). The N400 in a semantic categorization task across 6 decades. *Electroencephalography and Clinical Neurophysiology/Evoked Potentials Section*, *108*(5), 456–471. https://doi.org/10.1016/S0168-5597(98)00023-9

Luck, S. J. (2012). Event-related potentials. In H. Cooper, P. M. Camic, D. L. Long, A. T. Panter, D. Rindskopf, & K. J. Sher (Eds.), *APA handbook of research methods in psychology, vol 1: Foundations, planning, measures, and psychometrics* (pp. 523–546). American Psychological Association. https://doi.org/10.1037/13619-028

Misra, M., & Holcomb, P. J. (2003). Event–related potential indices of masked repetition priming. *Psychophysiology*, *40*(1), 115–130. https://doi.org/10.1111/1469-8986.00012

Polich, J. (2007). Updating P300: An integrative theory of P3a and P3b. *Clinical Neurophysiology*, *118*(10), 2128–2148. https://doi.org/10.1016/j.clinph.2007.04.019

Rugg, M. D. (1990). Event-related brain potentials dissociate repetition effects of high-and low-frequency words. *Memory & Cognition*, *18*(4), 367–379. https://doi.org/10.3758/BF03197126

Stalpaert, J., Cocquyt, E.-M., Miatton, M., Sieben, A., Van Langenhove, T., van Mierlo, P., & De Letter, M. (2021). A case series of verbal semantic processing in primary progressive aphasia: Evidence from the N400 effect. *International Journal of Language & Communication Disorders*, *56*(6), 1165–1189. https://doi.org/10.1111/1460-6984.12658

van Petten, C., Kutas, M., Kluender, R., Mitchiner, M., & McIsaac, H. (1991). Fractionating the word repetition effect with event-related potentials. *Journal of Cognitive Neuroscience*, *3*(2), 131–150. <https://doi.org/10.1162/jocn.1991.3.2.131>
